# Transcriptome Analysis of *Melocactus glaucescens* (Cactaceae) Reveals Metabolic Changes During *in vitro* Shoot Organogenesis Induction

**DOI:** 10.3389/fpls.2021.697556

**Published:** 2021-08-20

**Authors:** Gabriela Torres-Silva, Ludmila Nayara Freitas Correia, Diego Silva Batista, Andréa Dias Koehler, Sheila Vitória Resende, Elisson Romanel, Daniela Cassol, Ana Maria Rocha Almeida, Susan R. Strickler, Chelsea Dvorak Specht, Wagner Campos Otoni

**Affiliations:** ^1^Plant Biology Department/Laboratory of Plant Tissue Culture II—BIOAGRO, Federal University of Viçosa (UFV), Viçosa, Brazil; ^2^Department of Agriculture, Federal University of Paraíba (UFPB), Bananeiras, Brazil; ^3^Institute of Biology, Federal University of Bahia (UFBA), Salvador, Brazil; ^4^Laboratory of Plant Genomics and Bioenergy, Department of Biotechnology, School of Engineering of Lorena, University of São Paulo, Lorena, Brazil; ^5^Department of Botany and Plant Sciences, University of California, Riverside, Riverside, CA, United States; ^6^Department of Biological Science, College of Science, California State University East Bay, Hayward, CA, United States; ^7^Computational Biology Center, Boyce Thompson Institute, Cornell University, Ithaca, NY, United States; ^8^Plant Biology Section and the L. H. Bailey Hortorium, School of Integrative Plant Science, Cornell University, Ithaca, NY, United States

**Keywords:** calmodulin, *de novo* assembly, next-generation sequencing, non-model species, RNA-Seq, *WIND1*

## Abstract

*Melocactus glaucescens* is an endangered cactus highly valued for its ornamental properties. *In vitro* shoot production of this species provides a sustainable alternative to overharvesting from the wild; however, its propagation could be improved if the genetic regulation underlying its developmental processes were known. The present study generated *de novo* transcriptome data, describing *in vitro* shoot organogenesis induction in *M. glaucescens*. Total RNA was extracted from explants before (control) and after shoot organogenesis induction (treated). A total of 14,478 unigenes (average length, 520 bases) were obtained using Illumina HiSeq 3000 (Illumina Inc., San Diego, CA, USA) sequencing and transcriptome assembly. Filtering for differential expression yielded 2,058 unigenes. Pairwise comparison of treated vs. control genes revealed that 1,241 (60.3%) unigenes exhibited no significant change, 226 (11%) were downregulated, and 591 (28.7%) were upregulated. Based on database analysis, more transcription factor families and unigenes appeared to be upregulated in the treated samples than in controls. Expression of *WOUND INDUCED DEDIFFERENTIATION 1* (*WIND1*) and *CALMODULIN* (*CaM*) genes, both of which were upregulated in treated samples, was further validated by real-time quantitative PCR (RT-qPCR). Differences in gene expression patterns between control and treated samples indicate substantial changes in the primary and secondary metabolism of *M. glaucescens* after the induction of shoot organogenesis. These results help to clarify the molecular genetics and functional genomic aspects underlying propagation in the Cactaceae family.

## Introduction

The ability to regenerate and form an entire plant from individual tissues or organs, or even from a single somatic cell, is the basis of micropropagation techniques and plant regeneration systems (Rocha et al., 2018). Owing to high multiplication rates over short periods and in reduced and sterile spaces, tissue cultures allow large-scale and rapid *in vitro* propagation and conservation of plant material (Pérez-Molphe-Balch et al., 2015).

In the Cactaceae family, carefully managed propagation methods are essential for the prevention of overharvesting and the promotion of sustainable production of endangered species that are prized in the ornamental horticultural trade. *In vitro* regeneration methods offer an alternative to conventional propagation, especially for slow-growing endangered species (Lema-Rumińska and Kulus, 2014; Goettsch et al., 2015; Pérez-Molphe-Balch et al., 2015). *Melocactus glaucescens* has a light green stem and a white cephallium, which confer high ornamental value. Under natural conditions, *M. glaucescens* reproduces sexually and does not ramify or produce lateral shoots unless the plant suffers some form of injury (Machado, 2009). Unlawful harvesting and degradation of its natural habitat pose a serious threat to this species; thus, protocols for *in vitro* shoot organogenesis of *M. glaucescens* have been developed to address the overharvesting of this species in the wild (Torres-Silva et al., 2018).

*In vitro* propagation of *M. glaucescens* remains a challenge because organogenesis in plant growth regulators (PGR)-free medium results in low numbers of shoots per explant. On the other hand, organogeneses in media with PGR have been shown to result in high proportions of shoots with morphological and/or physiological alterations (Torres-Silva et al., 2018). Despite the observation of a somaclonal variation in the first round of shoot organogenesis by Torres-Silva et al. (2018), there is no correlation between this somaclonal variation and the observed morphological changes; therefore, further studies are necessary to improve the *in vitro* shoot production protocols of this species. Recent improvements to *in vitro* shoot production based on intentional wounding in the axillary meristems have successfully increased the number of shoots per explant (Torres-Silva et al., 2021). Availability of a transcriptome profile would expand the understanding of the molecular mechanisms involved in the development and physiology of this species and enable the use of molecular tools to improve *in vitro* propagation.

Transcriptome data offer an efficient way to discover genes or gene families encoding enzymes or transcription factors involved in various morphophysiological pathways (Xiao et al., 2013; Nadiya et al., 2018; Ebenezer et al., 2019), thus providing a valuable resource for the molecular characterization of biosynthetic pathways and gene regulatory networks involved in plant development (Pal et al., 2018). Nevertheless, transcriptome analysis remains relatively unexplored in most non-model plants. To date, few transcriptome studies of Cactaceae have been performed (Ibarra-Laclette et al., 2015; Qingzhu et al., 2016; Rodriguez-Alonso et al., 2018; Li et al., 2019; Xu et al., 2019), and none have looked into *in vitro* propagation and regeneration in this family.

The molecular bases of the processes underlying organogenesis are conserved through plant evolution (Ikeuchi et al., 2016); however, much less is known about the particulars of these processes in several plant species, among them, cacti. The goal of this study was to characterize changes in gene expression following *in vitro* shoot organogenesis in the non-model species *M. glaucescens*. The characterization of the *M. glaucescens* gene regulatory networks offers new insights into the physiological mechanisms that trigger regeneration in cacti that do not naturally emit branches. Additionally, this work provides useful information about the developmental patterns and processes of vegetative growth in Cactaceae in general.

## Materials and Methods

### Plant Material

**Figure 1:**
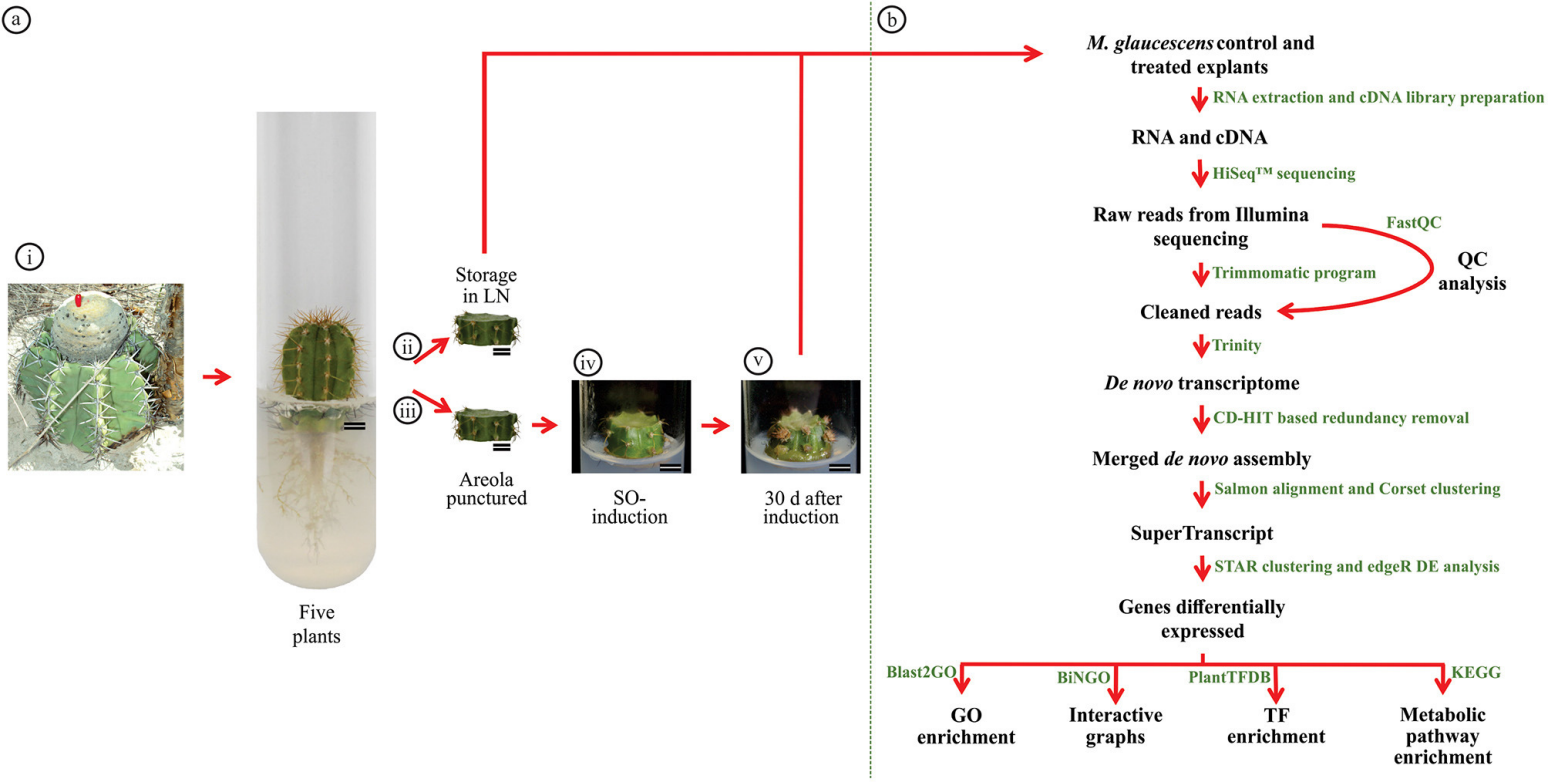
*Melocactus glaucescens* tissues are used for transcriptome analysis and workflow of transcriptome assembly and characterization. **(a)** Collection of samples: (i) seeds were collected from a natural population of *M. glaucescens* (Morro do Chapéu, Bahia, Brazil); (ii) following germination, control explants were stocked in liquid nitrogen immediately after excision; (iii) using the same plant donor, explants had their areola regions punctured three times with 0.18 × 8 mm needles and were then placed on MS full-strength medium supplemented with 17.76 μM benzyladenine and 1.34 μM naphthalene acetic acid to induce shoot organogenesis (SO); (iv) 30 days after SO induction, treated samples were stocked in liquid nitrogen (LN) until RNA extraction. **(b)** Transcriptome analysis pipeline and strategy used for *de novo* assembly and characterization.

Plant material for all analyses was obtained from *M. glaucescens* seeds germinated *in vitro*. The seeds were collected in February 2016 from mature individuals with a well-developed cephalium that were grown in Morro do Chapéu City (11°29′38.4" S; 41°20′22.5" W), Bahia State, eastern Brazil (Figure 1ai). In *M. glaucescens*, the apical meristem takes about 10 years to differentiate into a reproductive meristem, giving rise to a region called the cephalium, from which the flowers and fruits emerge (Machado, 2009). The population was identified and georeferenced as previously described by Lambert et al. (2006). A voucher specimen was deposited at the Herbarium of the Universidade Estadual de Feira de Santana, located in the municipality of Feira de Santana, Bahia State (Lambert et al., 2006). The plant material used in this study was identified by Dr. Sheila Vitória Resende (UFBA, Bahia, Brazil). Collection and access to genetic heritage strictly followed current Brazilian biodiversity legislation and was officially permitted by the Brazilian National System for the Management of Genetic Heritage and Associated Traditional Knowledge (SISGEN) under permission number A93B8DB. This species is endemic to the Bahia state and is listed as endangered by the Convention on International Trade in Endangered Species of Wild Fauna and Flora (UNEP-WCMC (Comps.), 2014) and the International Union for Conservation of Nature (IUCN) Red List of Threatened Species (Braun et al., 2013).

The seeds were disinfected with 96% ethanol for 1 min, 2% NaOCl commercial bleach (2.5% active chlorine; SuperGlobo®, Contagem, Minas Gerais, Brazil) for 10 min, and subsequently washed three times in sterile water under aseptic conditions. The seeds were then germinated in 500-ml glass flasks with rigid polypropylene lids (TC-003-2012; Ralm®, São Bernardo do Campo, São Paulo, Brazil), containing 50 ml of Murashige and Skoog (MS) culture medium (Murashige and Skoog, 1962) at quarter-strength concentration, supplemented with 15 g L^−1^ sucrose, and solidified with 7 g L^−1^ agar (A296 Plant TC; PhytoTechnology Lab®, Shawnee Mission, KS, USA) with pH 5.7 and autoclaving at 120°C, 1.5 atm for 20 min. Cultures were maintained at 25 ± 3°C under two fluorescent lamps (HO TLT; Sylvania, São Paulo, Brazil) with photosynthetically active radiation of 60 μmol m^−2^ s^−1^ (assessed by a portable LI-250A Light Meter device coupled with an LI-190R Quantum Sensor (LI-COR®, Lincoln, NE, USA) for a 16/8-h light/dark photoperiod.

Plants germinated *in vitro* for 54 weeks had their apical stem segments removed and were sectioned transversely, generating explants of 3–4 mm in height, according to previously established protocol by Torres-Silva et al. (2018). One explant was stocked in liquid nitrogen immediately after excision so it could be used as a control in comparative transcriptomics (Figure 1aii). A second explant from the same individual was punctured three times in the areola region with 0.18 × 8 mm needles (DBC132; Dong Bang Acupuncture Inc., Chungnam, South Korea) to initiate shoot organogenesis (Figure 1aiii) and placed in a vertical position inside glass tubes containing 15 ml of MS full-strength medium supplemented with 17.76 μM of benzyladenine (Sigma-Aldrich, St. Louis, MO, USA) and 1.34 μM of naphthalene acetic acid (Sigma-Aldrich) (Figure 1aiv). The tubes were sealed using rigid polypropylene lids. Cultures were maintained at 25 ± 3°C under 2 fluorescent lamps (Sylvania HO TLT) with photosynthetically active radiation of 60 μmol m^−2^ s^−1^ and a 16/8-h light/dark photoperiod. After 30 days of shoot organogenesis induction, five explants exhibiting shoot formation (Figure 1av) were selected for further analysis, constituting five biological replicates.

### RNA Isolation

Tissues from control and treated explants were ground in liquid nitrogen and total RNA was extracted with Tris®-Reagent (Sigma-Aldrich, St. Louis, MO, USA) according to the instructions of the manufacturer (Figure 1b). Briefly, 500 μl of Tris®-Reagent and 50 μl of chloroform: isoamyl alcohol (24:1) were added to 500 mg of the frozen tissue. The mixture was vortexed, stored on ice for 5 min, and centrifuged at 12,000 × *g* for 15 min at 4°C. The aqueous phase was decanted into a new microtube, and an equal volume of isopropanol was added for RNA precipitation. After incubation for 2 h at −20°C, the microtube was centrifuged again at 12,000 × *g* for 30 min at 4°C. The pellet was washed with 1 ml of 70% ethanol, dried, and eluted in diethyl pyrocarbonate water (Sigma-Aldrich).

### Library Preparations and RNA Sequencing (RNA-Seq)

Total RNA and Dynabeads® Oligo (dT) 25 (Thermo Fisher Scientific, Waltham, MA, USA) were used to isolate mRNA. The resulting mRNA fragments of ~400 nucleotides were converted to double-stranded complementary DNA (cDNA) using random hexamer primers and corresponding enzymes in accordance with standard DNA library protocols for sequencing. Briefly, cDNA was end-repaired, phosphorylated, and adenylated. Common TruSeq adapters containing 8-bp indexes (i5 and i7) suitable for Illumina sequencing were then ligated to the adenylated molecules, and the resulting libraries were amplified by 13 cycles of PCR to enrich for properly ligated molecules (Figure 1b). The final libraries were quantified using PicoGreen (Thermo Fisher Scientific) and equally combined into a single sample, which was then sequenced on an Illumina HiSeq 3000 (Illumina Inc., San Diego, CA, USA) instrument. Paired-end reads with an average length of 100 bp were obtained. Library preparation and sequencing were carried out by RAPiD Genomics, LLC (Gainesville, FL, USA).

### *De novo* Transcriptome Reference Assembly

RNA sequencing reads were processed using the Trimmomatic v0.36, with a sliding window of 4:25 and a minimum length of 50 (Bolger et al., 2014) to remove adaptor sequences, short reads, and low-quality reads. This resulted in clean paired-end reads and unpaired reads devoid of their partner sequences. FastQC (Andrews, 2010) was used before and after cleaning to check reads quality. Low-quality reads (Phred scores <20) were removed using Fastq_clean (Zhang et al., 2014). The clean reads, thus, obtained were then used to assemble the *de novo* transcriptome in Trinity v2.5.1 according to the following parameters: Trinity—seqTypefq—Left file_L003_1P, file_L004_1P—right file_L003_2P, file_L004_2P—CPU 60—output FILE_trinity_out —max_memory 100G (Grabherr et al., 2011) (Figure 1b).

### Construction of a SuperTranscript

To determine differential expression between control and treated tissues of *M. glaucescens*, a single, nonredundant SuperTranscript contig representing all isoforms was produced. Briefly, all reads from both treatments were collapsed into a single file using CD-HIT-EST v4.7 (Li and Godzik, 2006), with a sequence identity cutoff (-c) of 0.98. After that, the reads from each treatment were aligned with the cd-hit fasta file and transcript abundance was quantified using Salmon v0.9.1 (Figure 1b).

In the next step, clusters were formed based on shared reads and expression data using Corset v1.07 (Davidson and Oshlack, 2014). Finally, the clusters were transformed into a single sequence (SuperTranscript) containing combined information from all isoforms (Davidson et al., 2017). Alignment and quantification of SuperTranscript sequences were performed using STAR v2.5.3a (Dobin et al., 2013). This allowed the identification of uniquely mapped reads, mismatch rate per base, number of reads mapped to multiple *loci*, and number of chimeric reads (Figure 1b).

### Differential Expression Analysis

The counts of SuperTranscript clusters generated by STAR were used for the differential expression analysis between control and treated *M. glaucescens* explants. The Bioconductor edgeR package v3.30.3 evaluates gene-wise dispersions through conditional maximum likelihood, which enables differential expression analysis for each gene based on conditioning toward total counts for a certain gene (Robinson et al., 2010). In this study, edgeR with fold change (log2) > 2 and *P* ≤ 0.05 was applied. Genes with count per million values >1 in at least two libraries were selected for differential expression analysis. The treated samples were compared to the control samples to determine upregulation and downregulation (Figure 1b).

BLASTx results obtained by searching against the National Center for Biotechnology Information (NCBI) nr database were imported into Blast2GO and Gene Ontology (GO) annotations, after which Enzyme Commission classifications were performed in Blast2GO (Conesa and Götz, 2008). Further functional analysis of the transcriptome assigned Kyoto Encyclopedia of Genes and Genomes (KEGG) Orthology terms to the transcripts and mapped them to KEGG pathways (Kanehisa et al., 2008) using the KEGG Automatic Annotation Server (KAAS) (http://www.genome.jp/tools/kaas/). Interactive graphs of downregulated and upregulated transcripts were generated in the Biological Networks Gene Ontology (BiNGO) tool (Maere et al., 2005), and the result was displayed using Cytoscape 3.4.0 (http://www.cytoscape.org) (Figure 1b).

Transcription factor information was obtained from the Plant Transcription Factor Database v4.0 (PlantTFDB) (http://planttfdb.cbi.pku.edu.cn/) (Jin et al., 2017). *M. glaucescens* downregulated and upregulated transcripts were subjected to BLASTx analysis against the PlantTFDB of *Beta vulgaris* (the closest organism whose genome is annotated in this platform) using the scoring matrix BLOSUM62 and an expected threshold of 0.1 (Figure 1b).

### Validation of Differentially Expressed Genes

Two genes were chosen for *M. glaucescens* transcriptome validation: *WOUND INDUCED DEDIFFERENTIATION 1* (*WIND1*) and *CALMODULIN* (*CaM*). Total RNA obtained from three control and three treated explants were used to synthesize single-stranded cDNAs. Total RNA (3 μg) and SuperScript^TM^ II First-Strand Synthesis System (Thermo Fisher Scientific) were used according to the recommendations of the manufacturer.

Sequences for the *WIND1, CaM*, and *GLYCERALDEHYDE 3-PHOSPAHTE DEHYDROGENASE* (*G3PDH*) primers were obtained from the *M. glaucescens* transcriptome (Table 1). The primers were designed using the NCBI Primer-BLAST (http://www.ncbi.nlm.nih.gov/tools/primerblast/index.cgi?LINK_LOC=BlastHome) with the following settings: primer melting temperature, 60°C; primer GC content, 50–60%; PCR product size, 100–200 bp. Real-time quantitative PCR (RT-qPCR) was performed on a CFX96 Touch™ Real-Time PCR Detection System (Bio-Rad, Hercules, CA, USA) using the qPCR-SYBR-Green mix/Rox (Ludwig Biotec®, Alvorada, Rio Grande do Sul, Brazil).

**Table 1:** Sequences of primers used to validate differential expression of the *Melocactus glaucescens* transcriptome.

**Primers**	**Sequence**
***GLYCERALDEHYDE-3-PHOSPHATE DEHYDROGENASE***	
Forward	5′-AAGGTCCAAGTAGCAAGGGC-3′
Reverse	5′-TGCACCGATGTCTCTTCCAC-3′
***WOUND INDUCED DEDIFFERENTIATION 1***	
Forward	5′-TGTTCAATCTCATCACCATTGC-3′
Reverse	5′-AGCCCATAACACTTGTCAGCA-3′
***CALMODULIN***	
Forward	5′- AAGGGTGGACAAAGGCGAAT-3′
Reverse	5′-CCTCCAGGTACATCGGAAACC-3′

All qPCR reactions were performed in duplicates for three biological replicates from each treatment. The total reaction volume of 10 μl included 4 μl of SYBR-Green, 1 μl (4 μM) of each primer, 3 μl of diethylpyrocarbonate-treated water, and 1 μl (40 ng) of the cDNA sample. Amplification conditions were as follows: 2 min at 50°C, 10 min at 95°C, followed by 40 cycles at 95°C for 16 s and 60°C for 60 s. The melting curve was obtained from 60 to 95°C at 0.1°C/s. The comparative cycle threshold method (2^−Δ*ΔCt*^) (Livak and Schmittgen, 2001) was used to calculate the fold-change of target genes.

## Results

### Illumina Sequencing and *de novo* Assembly of the *M. glaucescens* Transcriptome

Changes in gene expression between *M. glaucescens* explants subjected (treated) to shoot organogenesis or not (controls) were investigated using Illumina HiSeq 3000 sequencing (Figure 1b). Initial data processing involved demultiplexing and trimming to eliminate Illumina adapter sequences (Figure 1b). Of the ~25 million processed reads thus generated, only a small percentage contained any Ns. There were no reads without a quality value that contained any contigs, and no chimeric sequences were detected by STAR (Table 2).

**Table 2:** Raw data and assembly statistics for transcriptomes from *M. glaucescens* explants.

**Description**	**Control**	**Treated**
Raw data statistics		
Total number of raw reads	11,778,019	13,371,985
Clean reads	4,712,559	5,433,394
Clean bases (Mb)	1.18	6.63
Assembly statistics		
Number of transcripts	2,231	12,247
Total transcript length (bp)	1,180,871	6,626,929
Transcript N50	527	513
Max transcript size (bp)	7,366	7,403
Mean transcript size (bp)	527	513
Min transcript size (bp)	201	201
GC content (%)	55	54
Unigenes with significant BLAST hits	1,447	1,796
Unigenes without significant BLAST hits	20	36

*Control samples were collected before and treated samples after de novo shoot organogenesis induction*.

A total of 2,231 assembled transcripts with an average length of 527 bp and an average GC content of 55% were obtained from control explants (Table 2). The maximum size of any assembled transcript was 7,366 bp, and the minimum size was 201 bp. Although substantially more abundant (12,247 in total), transcripts obtained from treated samples exhibited similar values to those from control samples, with an average length of 513 bp, a GC content of 54%, and maximum and minimum sizes of 7,403 and 201 bp, respectively (Table 2).

### Differential Expression Analysis

**Figure 2:**
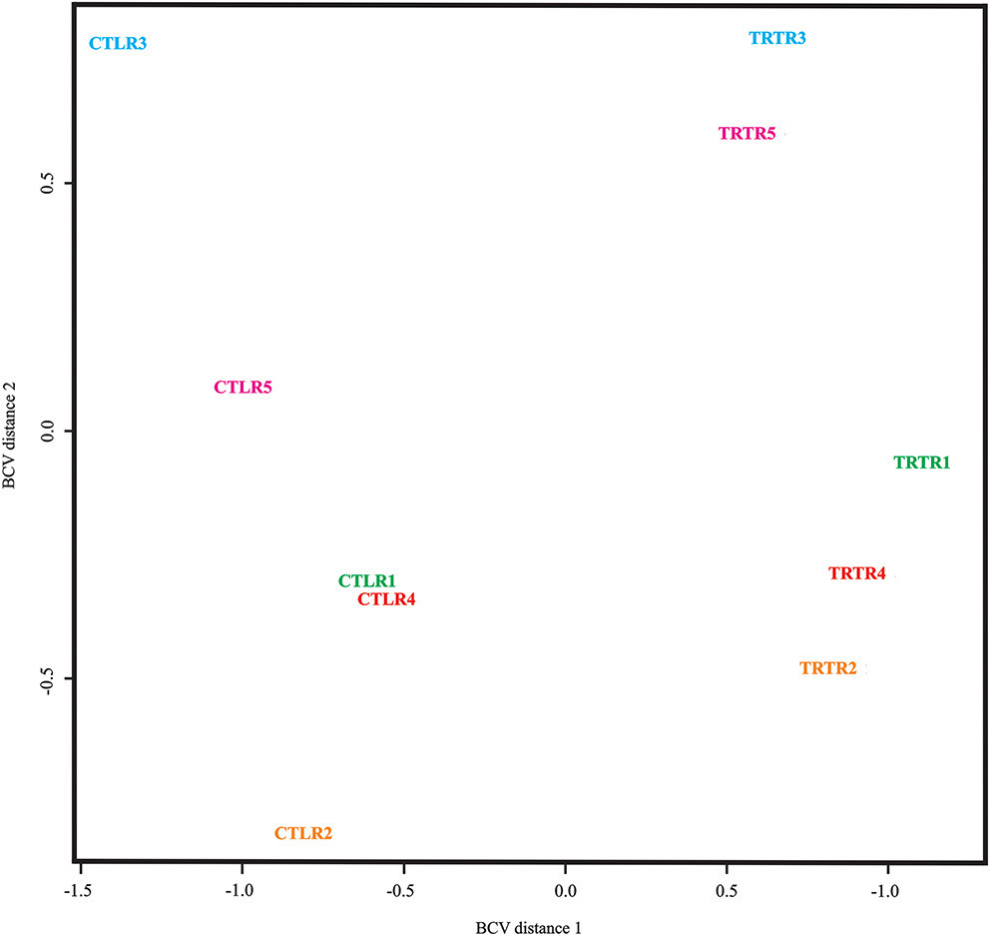
Multidimensional scaling relationship between *M. glaucescens* explants before (CTL) and after (TRT) shoot organogenesis induction. In the plot, the biological coefficient of variation (BCV) dimension 1 separates control and treated samples; whereas BVC dimension 2 separates the genotypes.

Initial sample processing for differential expression analysis separated the samples based on multidimensional scaling using the biological coefficient of variation (BCV). As shown by the plot in Figure 2, control and treated samples were separated by BCV distance 1, and genotypes were separated by BCV distance 2. Accordingly, control and treated samples from plants 3 and 5 clustered in the upper part of the plot; whereas those from plants 1, 2, and 4 were localized to the lower part of the plot.

**Figure 3:**
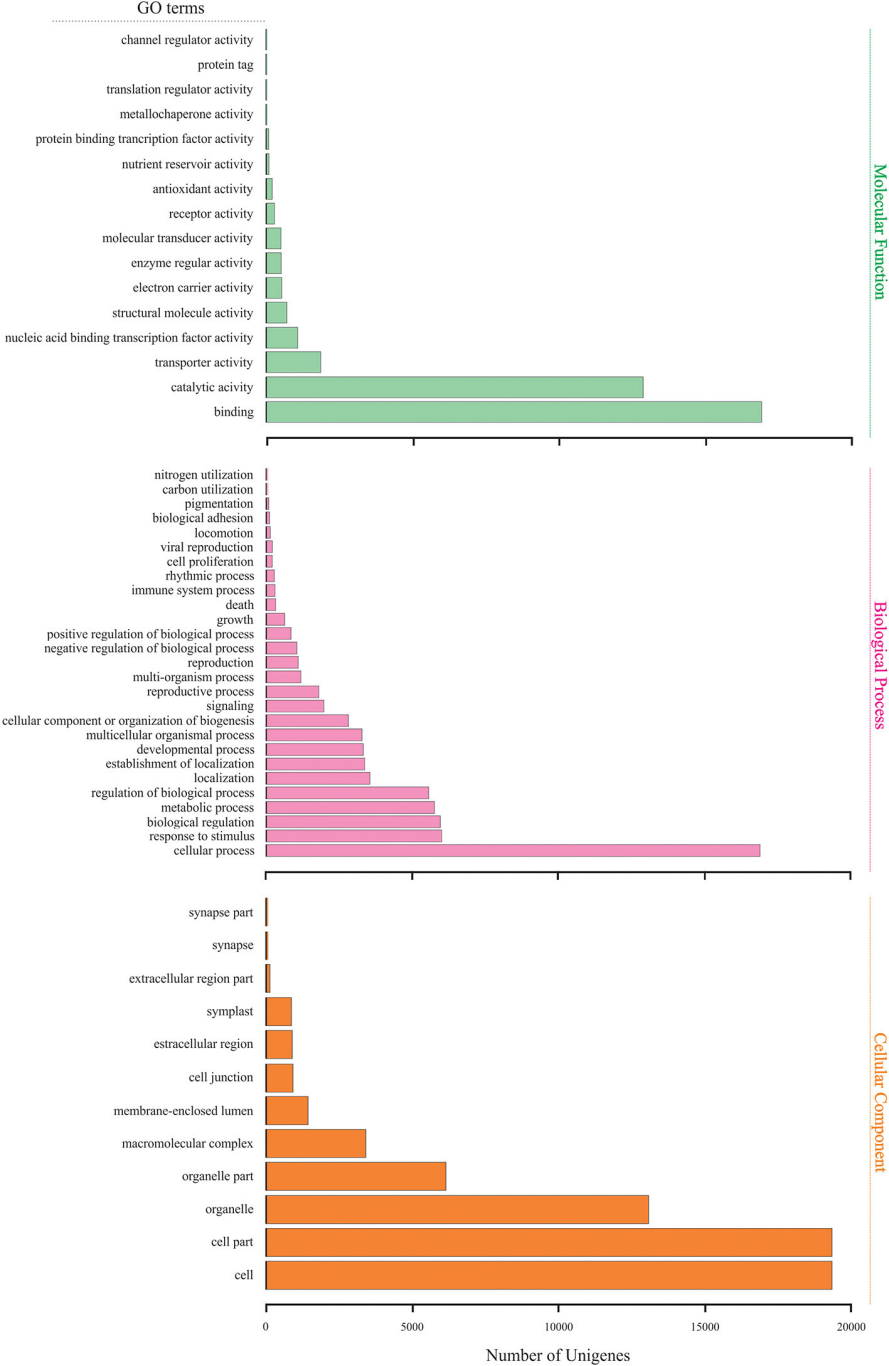
Gene Ontology annotation for all assembled unigenes in the *M. glaucescens* transcriptome.

The merged transcriptome was functionally annotated in Blast2GO by importing the BLASTx comparison of *M. glaucescens* contigs against the NCBI nr database. The output was applied to GO mapping and functional characterization. Transcripts were grouped into three main GO categories: “molecular function,” “biological processes,” and “cellular component” (Figure 3). In the “molecular function” category, catalytic activity and binding were the prevalent groups. In the “biological process” category, cellular processes were the most abundant group, followed by regulation of biological processes, metabolic processes, biological regulation, and responses to stimuli. In the “cellular component” category, cells, cell parts, and organelles were the predominant groups.

**Figure 4:**
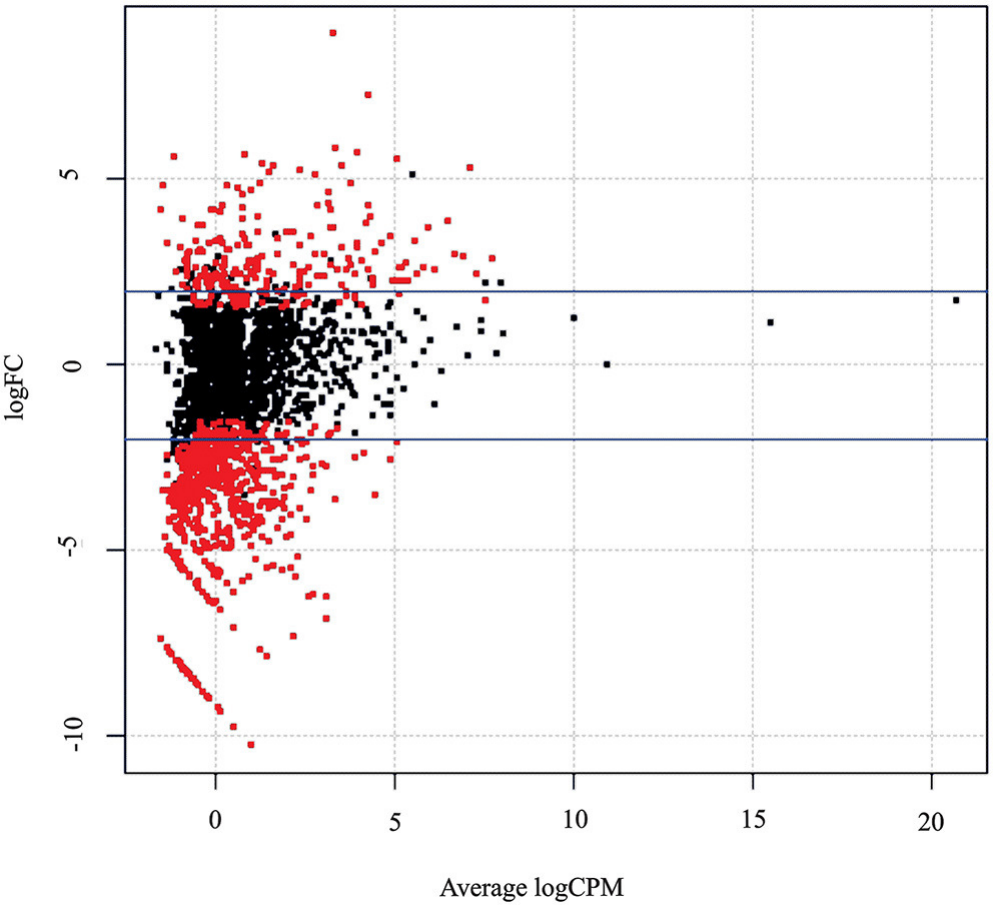
Differentially expressed genes in *M. glaucescens* explants before (control) or after (treated) shoot organogenesis induction. Blue lines indicate genes with no significant changes in expression (1,241 genes); whereas red dots indicate genes that were either significantly downregulated (226) or upregulated (591) in treated explants. The false discovery rate was set at *P* ≤ 0.05. FC, fold change; CPM, counts per million mapped reads.

After applying the low expression filter, a total of 2,058 unigenes were identified in the *M. glaucescens* transcriptome. Differential expression profiles of control and treated *M. glaucescens* explants identified sets of upregulated and downregulated unigenes (Figure 4). Pairwise comparison of treated vs. control samples revealed that 1,241 unigenes (60.3%) displayed no significant change in expression, 226 (11%) were downregulated, and 591 (28.7%) were upregulated in treated explants (Supplementary Material 2).

**Figure 5:**
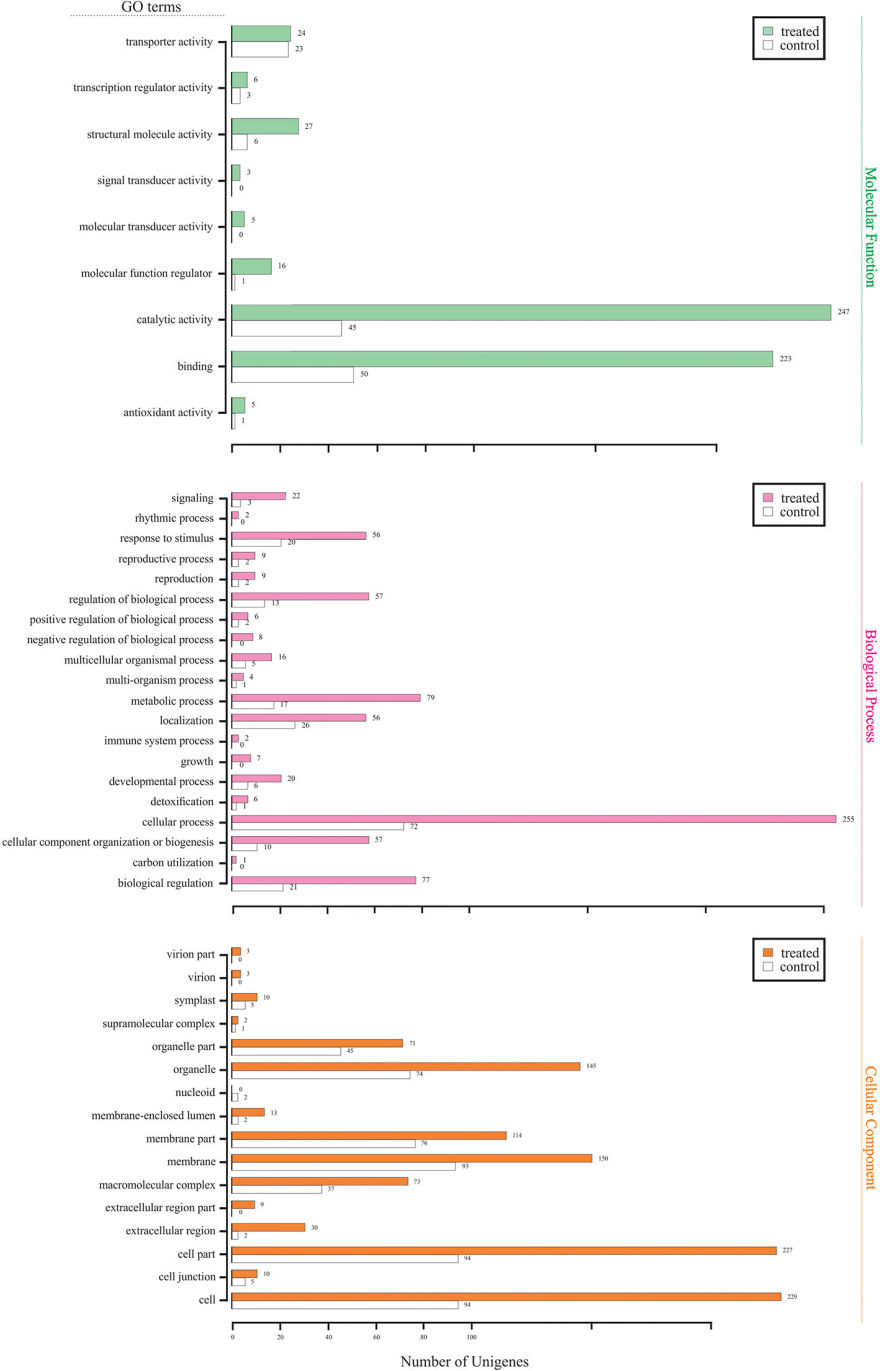
Gene Ontology functional profile in *M. glaucescens* explants before (control) and after (treated) shoot organogenesis induction.

Further analysis categorized the differentially expressed unigenes into 44 functional classes, with the most frequent groups in the “molecular function” category being catalytic activity (247 unigenes) and binding (223 unigenes). The predominant group in the “biological process” category was cellular processes (255 unigenes). In the “cellular component” category, cells (229 unigenes) and cell parts (227 unigenes) were the most abundant. Finally, treated samples were characterized by more unigenes in almost all GO groups and GO categories (Figure 5).

### Pathway Mapping Using KEGG and BiNGO

The KAAS was employed to map transcripts to their biological pathways. A bi-directional best hit scheme was employed for the KEGG Orthology assignments with a default best-hit rate > 0.95. KEGG pathway mapping of the downregulated or upregulated *M. glaucescens* genes identified 748 unigenes assigned to 233 KEGG pathways (Supplementary Material 1 and Table 2). Downregulated and upregulated transcripts were categorized into distinct KEGG pathways, indicating that shoot organogenesis induction played a specific role in cacti metabolism.

Some KEGG pathways, such as amino acid metabolism and ribosome, were observed in both treated and control samples, but the transcripts were not identical (indicated with ^*^). This suggests that these pathways were rewired to meet the metabolic demands of shoot organogenesis. Few KEGG pathways presented only downregulated transcripts (i.e., photosynthesis and antenna proteins), indicating the photoautotrophic growth of control samples (Supplementary Material 1 and Table 2). Upregulated transcripts shared KEGG pathways related to transcription, signaling, cell cycle, cytoskeletal rearrangement, cell differentiation, and mitochondrial dysfunction (Supplementary Material 1).

**Figure 6:**
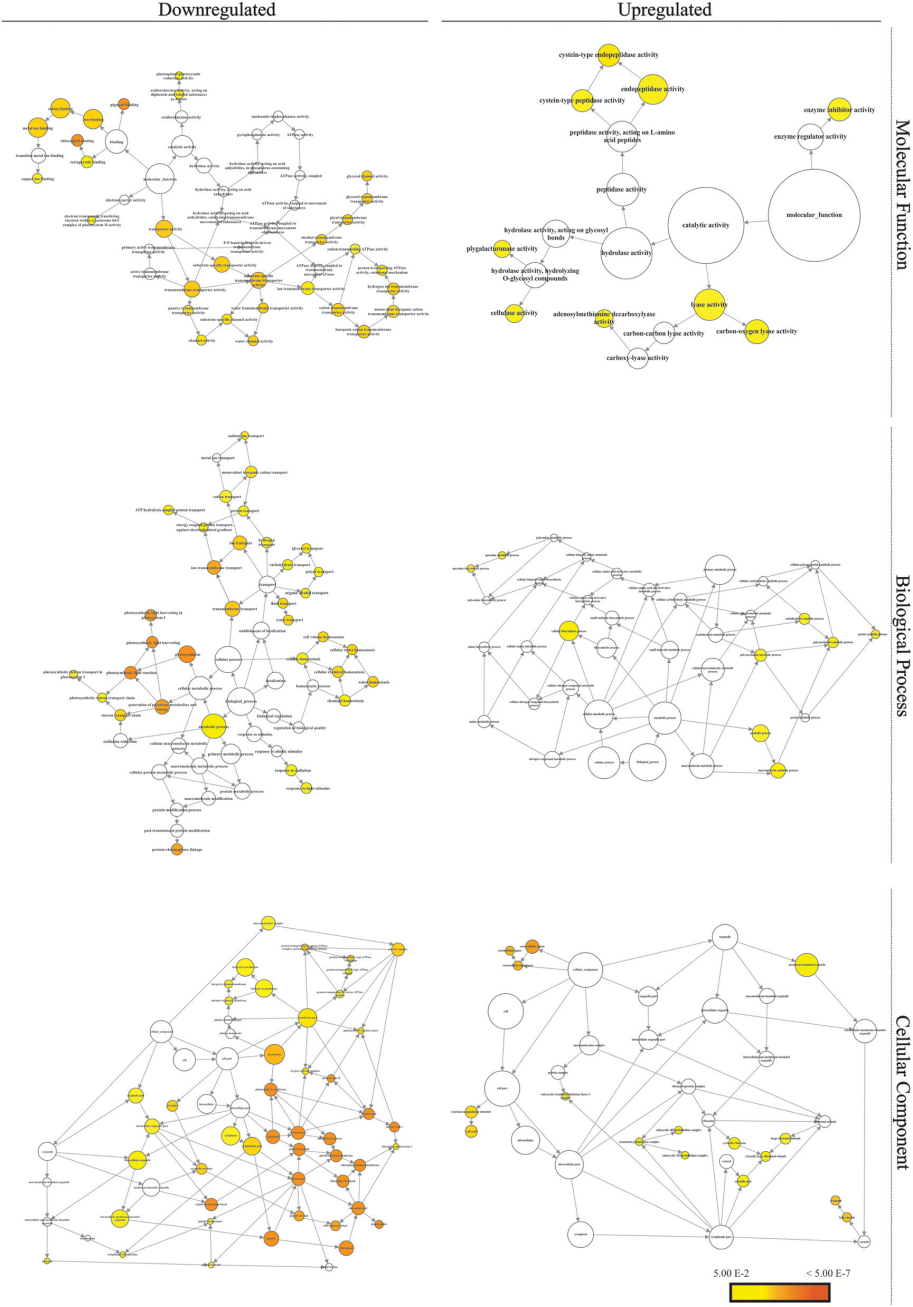
Networks of differentially expressed genes. The networks were developed using GO categories in BiNGO for *M. glaucescens* explants before (control) and after (treated) shoot organogenesis induction. Bubble size and color indicate the frequency of the GO term and the *P*-value, respectively.

Biological Networks Gene Ontology analysis of the GO categories assigned to the *M. glaucescens* transcriptome identified three major GO categories among the upregulated unigenes (Figure 6). Overall, the downregulated unigenes displayed a dispersed pattern, with smaller and more numerous bubbles than the upregulated unigenes. In contrast, the latter were more concentrated in certain “biological process” categories, such as spermine biosynthetic and metabolic processes, pectin catabolic processes, polysaccharide metabolic and catabolic processes, and carbohydrate catabolic processes. The most abundant groups in the “cellular component” category were cell wall, extracellular region, ribosome, and vacuole. In the “molecular function” category, the most abundant groups corresponded to catalytic activity (lyase and hydrolase activities) and enzyme regulator activity.

### Analysis of Transcription Factors

Multigene transcription factor families play significant roles in the regulation of gene expression during plant development and metabolic processes. BLASTx of the identified downregulated and upregulated unigenes against the *B. vulgaris* transcription factor database allowed the identification of 177 transcription factors from 39 families. The most abundant family was C3H (20), followed by LBD (16), C2H2 (11), WRKY (10), HB-other (9), ERF (9), HSF (8), and bHLH (7). Remarkable differential expression was observed among *M. glaucescens* unigenes owing to the downregulation of seven and upregulation of 16 transcription factor family members (Table 3).

**Table 3:** Transcription factor families containing downregulated and upregulated unigenes following *M. glaucescens* shoot organogenesis induction.

**Transcription factor family**	**Domain**	**No. of unigenes**	
		**Downregulated**	**Upregulated**
ERF family protein	AP2	2	7
AP2 family protein	AP2	1	0
Dof family protein	zf-Dof	0	4
LBD family protein	DUF260	5	11
ARF family protein	B3 and auxin response	1	5
B3 family protein	B3	0	2
LSD family protein	zf-LSD1	2	2
G2-like family protein	G2-like	2	3
GRAS family protein	GRAS	1	3
HD-ZIP family protein	Homeobox	0	1
HB-other family protein	Homeobox	4	5
TALE family protein	Homeobox	0	3
C3H family protein	zf-CCCH	3	17
NAC family protein	NAM	1	5
bZIP family protein	bZIP_1	4	2
WRKY family protein	WRKY	0	10
MYB family protein	Myb_DNA-binding	1	4
MYB_related family protein	Myb_DNA-binding	0	4
bHLH family protein	HLH	4	3
EIL family protein	EIN3	0	2
HSF family protein	HSF_DNA-bind	1	7
C2H2 family protein	zf-C2H2	3	8
Trihelix family protein	Trihelix	0	5
GATA family protein	GATA	0	2
E2F/DP family protein	E2F_TDP	0	2
CAMTA family protein	CG-1	0	1
NF-YC family protein	NF-YC	0	1
NF-YA family protein	CBFB_NFYA	1	0
NF-X1 family protein	zf-NF-X1	3	1
ZF-HD family protein	ZF-HD_dimer	1	0
SBP family protein	SBP	0	1
YABBY family protein	YABBY	0	1
FAR1 family protein	FAR1	0	1
CO-like family protein	zf-B_box and CCT	1	1
MIKC_MADS family protein	SRF-TF and K-box	0	1
GRF family protein	WRC and QLQ	1	0
S1Fa-like family protein	S1FA	1	0
TCP family protein	TCP	5	0
DBB family protein	zf-B_box	1	0

### Validation of Specific Gene Expression Profiles

**Figure 7:**
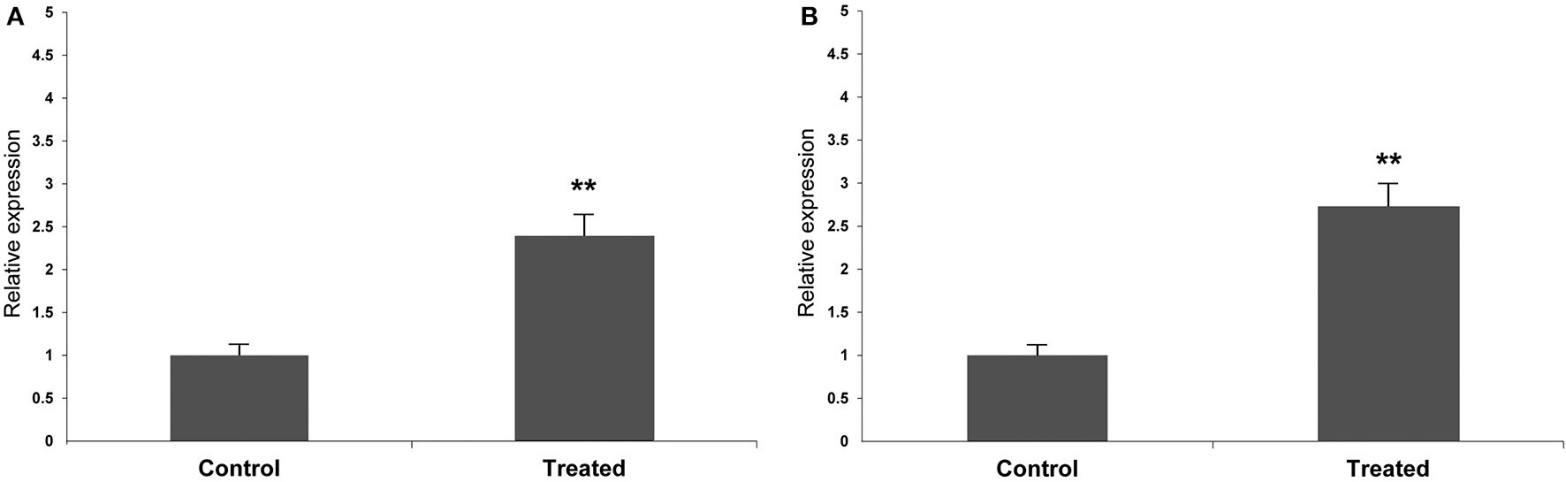
Real-time quantitative PCR (RT-qPCR) expression profile of target genes in *M. glaucescens* explants before (control) and after (treated) shoot organogenesis induction. **(A)** *WOUND INDUCED DEDIFFERENTIATION 1 (WIND1)*. **(B)** *CALMODULIN (CaM)*. **P ≤ 0.05.

To validate candidate genes obtained from comparative transcriptome analysis, RT-qPCR was performed on *WIND1* and *CaM* as targets, and *G3PDH* as internal reference genes, in control and treated explants. The expression patterns of *WIND1* and *CaM* were consistent with those obtained by transcriptome analysis (Figure 7), confirming the reliability of the transcriptome data.

## Discussion

This is the first study to explore the application of RNA-Seq data for the analysis of transcript levels following somatic organogenesis induction in an ornamental cactus. *M. glaucescens* is not a model species and lacks a sequenced genome. A major challenge when analyzing non-model species is that many transcripts cannot be annotated because they are too divergent from the model species to be identified (Garg and Jain, 2013; Brereton et al., 2016).

Given that the samples were derived from seeds, genetic diversity among biological replicates was as important as the experimental treatments in defining transcriptomic differences. This was noted in the morphogenetic response calculated using BCV dimensions, which compared treatment vs. genotype (Figure 2). Genotype variability may explain the difficulty reported by Torres-Silva et al. (2018) in finding a relationship between morphological alterations and somaclonal variation during *in vitro* shoot production of *M. glaucescens*.

Changes in GO categories also reflect the large-scale reorganization that treated explants undergo during regeneration (Zhao et al., 2008). Genes related to the mitochondria, cell wall, endoplasmic reticulum, cell organization, and biogenesis were upregulated during shoot organogenesis induction. This upregulation is likely a consequence of the increased protein synthesis necessary to support cell division and cell wall formation during regeneration (Bao et al., 2009). Furthermore, changes in cell fate can occur as a result of a new balance between euchromatin and heterochromatin following dynamic alterations in chromatin structure (Zhao et al., 2008), indicated here by the presence of histone-encoding genes among the upregulated transcripts (Figure 5).

Transcription factor families and unigenes were more abundant among the upregulated than downregulated unigenes (Table 3). This finding indicates that many metabolic pathways are activated during shoot organogenesis induction. The families of transcription factors found to be upregulated in this study (i.e., NF-Y, MYB, ERF, E2F, LBD, and NAC) are akin to those identified during plant organogenesis, further confirming the role of these regulators in vegetative regeneration (Cheong et al., 2002; Bao et al., 2009; Cervantes-Pérez et al., 2018; Nadiya et al., 2018; Pal et al., 2018; Xi et al., 2019).

During the *in vitro* culture preceding shoot organogenesis induction, the metabolism of *M. glaucescens* was characterized by fewer upregulated than downregulated genes, corroborating the low growth rate typical of cacti cultures (Lema-Rumińska and Kulus, 2014; Pérez-Molphe-Balch et al., 2015). Downregulated genes were largely involved in primary metabolism. They included transcripts related to photosynthesis (15) and antenna proteins (5) identified in KEGG pathways (Supplementary Material 1 and Table 2) and a large number of photosynthesis-related bubbles observed in the BiNGO graphs (Figure 6).

*In vitro* plant tissue cultures are established in closed culture vessels to control microbial contamination; however, this limits CO_2_ availability and requires the addition of an exogenous carbon source (e.g., sucrose) to the medium (Batista et al., 2018). As observed previously in *Euphorbia characia*, when sucrose is no longer available in the culture medium, photosynthetic carbon fixation is reestablished. This physiological adaptation to environmental changes (Hardy et al., 1987) allows the cultures to grow photoautotrophically. The results suggest a possible metabolic switch from photoautotrophy to photoheterotrophy when explants were subjected to shoot organogenesis, with the downregulation of chloroplast/plastid unigenes (Figure 6). Similar changes in gene expression have been observed in the regeneration of *Populus* and *Agave salmiana* and during the flower induction of *Hylocereus polyrhizus*, indicating that many morphogenic processes elicit a common pattern of metabolic changes (Bao et al., 2009; Cervantes-Pérez et al., 2018; Xiong et al., 2020). Additionally, the downregulation of *OXYGEN-EVOLVING ENHANCER* (Mayfield et al., 1987) and *EARLY LIGHT-INDUCED* (Hutin et al., 2003) homologs in the dataset (*P* ≤ 0.05) suggests photo-oxidative stress, which is probably caused by the *in vitro* conditions of these cultures (Batista et al., 2018).

The *TEOSINTE BRANCHED1-CYCLOIDEA-PROLIFERATING CELL FACTOR* (TCP) family was one of the few transcription factor families to be downregulated (*P* ≤ 0.05) rather than upregulated following shoot organogenesis induction (Table 3). TCPs play an important role in pattern formation through the suppression of ectopic meristem generation. Meristem formation is precluded by the expression of regulators that suppress the expression of *CUP-SHAPED COTYLEDON* genes (Koyama et al., 2010). Other targets of TCP suppression are miR164, *ASYMMETRIC LEAVES1* (*AS1*), *INDOLE-3-ACETIC ACID3/SHORT HYPOCOTYL2* (*IAA3/SHY2*), and *SMALL AUXIN UPREGULATED RNAs* (*SAUR*) (Ikeda and Ohme-Takagi, 2014). Some of the genes (or their targets) were suppressed by TCP and were upregulated during *M. glaucescens* shoot organogenesis induction (*P* ≤ 0.05); they included NAC family proteins (miR164 target) and *SAUR* genes (Supplementary Material 2 and Table 3). Accordingly, the TCP family may play a significant role in determining the absence of branching in *M. glaucescens*. Thus, this topic may be an important target for future studies aimed at improving shoot organogenesis induction in cacti that do not naturally emit lateral branches.

Furthermore, we report that plant hormone signal transduction pathways were altered during shoot organogenesis in *M. glaucescens*. KEGG analysis revealed that the upregulated transcripts included nine unigenes related to auxins (auxin response protein IAA, auxin-responsive *GH3* gene family, and *SAUR* family), gibberellins (DELLA protein), abscisic acid (abscisic acid receptor PYR/PYL family), ethylene (EIN3-binding F-box protein and ethylene-insensitive protein 3), and brassinosteroids (BR-signaling kinase and protein brassinosteroid insensitive 2). This finding indicates that stem growth, cell elongation, and cell division pathways are significantly activated during shoot organogenesis induction (Ikeuchi et al., 2019).

Interestingly, most *GIGANTEA (GI)-CONSTANS (CO)-FLOWERING LOCUS T* (*FT*) pathway genes appeared upregulated in this study. Following *M. glaucescens* shoot organogenesis induction, GI likely acted as an activator of *CO*, which in turn activated *FT*. Although *FT* was not significantly upregulated in the dataset, *FT INTERACTING PROTEIN 1* (*FTIP1*) was overexpressed (*P* ≤ 0.05). It has been proposed that members of the *FTIP* family regulate the trafficking of SHOOT MERISTEMLESS (STM) proteins, determining the appropriate balance between stem cell populations and their differentiation into lateral organs (Liu et al., 2012).

Several abscisic acid-related genes were also upregulated in the dataset (*P* ≤ 0.05). It has been suggested that abscisic acid modulates *GI* signaling and is required for drought escape responses. In this respect, abscisic acid signaling provides a link between osmotic stress and shoot organogenesis (Huang et al., 2012). Besides contributing to the stress response, *GI* is involved in circadian rhythm processes (Montaigu et al., 2014). Moreover, it has been suggested that *GI* and *FT*, together with *SEPALLATA* (*SEP*) and *FRIGIDA* (*FRI*), participate in the early flowering of white lupin (Rychel et al., 2019). We also found that *FRI* was upregulated in the dataset (*P* ≤ 0.05), pointing to circadian rhythm regulation during *M. glaucescens* shoot organogenesis induction.

Another set of upregulated genes included various *LIGHT-DEPENDENT SHORT HYPOCOTYLS* (*LSH*) homologs (*P* ≤ 0.05). Previous studies (Takeda et al., 2011; Bencivega et al., 2016) detected *LSH3* and *LSH4* in organ boundary cells. *LSH4* modulates auxin signaling or transport and dictates the orientation of cell division (Bencivega et al., 2016). *LSH* expression is directly regulated by *CUP-SHAPED COTYLEDON1* (*CUC1*) and *CUC2* (Takeda et al., 2011), although these genes were not significantly upregulated in the dataset (*P* ≤ 0.05).

Auxin controls most plant developmental responses and is potentially involved in *M. glaucescens* shoot organogenesis. For instance, Aux/IAAs and *TOPLESS/TOPLESS-RELATED* (*TPL/TPR*) genes were upregulated in the induced dataset (*P* ≤ 0.05), together with various ubiquitin-related (E1-, E2-, and E3-related genes) and F-box genes, especially *SKP-like* genes. In the absence of auxin, Aux/IAAs facilitate interactions between *AUXIN RESPONSE FACTORS* (*ARF*) and *TPL* co-repressors, inhibiting the expression of auxin-inducible genes. In contrast, in the presence of auxin, Aux/IAAs interact with F-box proteins, such as *SKP, RBX, CUL*, and *TIR1/AFB*, leading to the activation of the ubiquitinoylation enzyme system that promotes Aux/IAA degradation and the transcriptional activation of auxin-inducible genes. The affinity of Aux/IAAs for their interaction partners sets the auxin response threshold and determines the sensitivity of cells to auxin (Leyser, 2018). *TORNADO2* (*TRN2*), which was also upregulated in the dataset (*P* ≤ 0.05), is involved in the basipetal transport of auxin (IAA). The latter, in turn, modulates growth, organ structure, and cell differentiation, and promotes the organization of the peripheral zone of the shoot apical meristem (Chiu et al., 2007).

Given the involvement of auxin in several cellular events, we expected to find unigenes belonging to the *SAUR* family among upregulated and downregulated genes (Supplementary Material 2 and Table 2). Most vascular plant species contain between 60 and 140 *SAUR* genes in their genomes (Stortenbeker and Bemer, 2018), encoding various small transcripts tasked with a rapid response to auxin. The corresponding proteins are related to auxin-induced cell elongation, which follows the acid growth theory (Stortenbeker and Bemer, 2018). The presence of *SAUR* unigenes in *M. glaucescens* indicates that auxin responses promoting cell elongation and growth may differ between shoot organogenesis induction and its absence. The detection of transcription factors responsible for cell elongation exclusively among downregulated unigenes (e.g., GRF family) further supports this hypothesis (Table 3).

*WOUND INDUCED DEDIFFERENTIATION 1* expression is related to the acquisition of regeneration competence in culture, and transcripts were found to be upregulated in *M. glaucescens* treated explants after 30 days of shoot organogenesis induction (*P* ≤ 0.05). This result was confirmed by RT-qPCR (Figure 7A).

Wound signaling is initiated immediately after damage, with cells in the vicinity of the wound displaying remarkable plasticity and then reprogrammed to meet urgent repair tasks (Xu, 2018; Shanmukhan et al., 2020). Initial changes reflect a rapid physical and chemical response to wounding; they involve alterations in plasma transmembrane potential and intracellular Ca^2+^ concentration, increase in apoplastic glutamate, and H_2_O_2_ generation (Choi et al., 2017; Toyota et al., 2018; Xu, 2018). In comparison, it takes hours to initiate regeneration responses (Shanmukhan et al., 2020). The increased activation of various genes, including stem cell regulators and the concomitant hormonal surge, was previously reported as a side effect of wounding. Indeed, pathways linking wound signaling, *WIND1* expression, and the production of plant hormones to promote regeneration have only recently been investigated (Ikeda and Ohme-Takagi, 2014; Ikeuchi et al., 2019, 2020; Shanmukhan et al., 2020; Ye et al., 2020).

Recent reports showed that one of the primary events that occur at the wound site is a burst of jasmonate, which is responsible for inducing the expression of AP2/ERF genes (such as *WIND1*). These, in turn, may play an important role in wound-induced auxin biosynthesis and trigger regeneration processes (Ikeuchi et al., 2020; Ye et al., 2020). In *M. glaucescens*, shoot organogenesis induction overlaps with the wounding response. This may be due to TCP-mediated suppression, which triggers the gene expression cascade leading to shooting organogenesis. Wounded explants of *M. glaucescens* expressed *WIND1* even 30 days after shoot organogenesis induction, demonstrating that *WIND1* might also be involved in long-term responses to wounding. Wounding may trigger pathways related to cell death repression (Lin et al., 2011), and the presence of *PEROXIDASE 9, PEROXIDASE 12*, and *CaM* among upregulated transcripts indicates that overall metabolism could be modulated by the wounding stimulus. In addition, the jasmonate burst has been correlated with *S-adenosyl methionine synthetase* (*SAM*) expression. *SAM* leads to ethylene production and induces a response to signals that also mediate polyamine synthesis. By extension, *SAM* may generate an auxin burst that alters the ratio between wall and membrane constituents. This, in turn, results in altered membrane fluidity and access to exogenous hormones, eventually affecting regeneration competence (Ikeuchi et al., 2019).

Plants employ Ca^2+^ as a versatile second messenger in response to abiotic and biotic stimuli (i.e., light, temperature, mechanical disturbance, drought, osmotic stress, plant hormones, and pathogen elicitors), and *CaM* is one of the main Ca^2+^ sensors in plants (Hashimoto and Kudla, 2011; Zeng et al., 2015). *CaM* transduces signals that generate specific or overlapping responses to increased cellular Ca^2+^ (Zeng et al., 2015). In line with its widespread role in morphophysiological processes, *CaM* was upregulated in *M. glaucescens* treated explants 30 days after shoot organogenesis induction (*P* ≤ 0.05), as validated also by RT-qPCR (Figure 7B).

In *Arabidopsis*, seven genes encode four *CaM* isoforms (CaM1/4, CaM2/3/5, CaM6, and CaM7), and the differences between them account for the varied interaction/activation of CaM targets by the diverse isoforms and *CaM*-related proteins (Ranty et al., 2006; Kushwaha et al., 2008). During shoot organogenesis induction of *M. glaucescens, CaM* was related to 16% of the KEGG pathways, including the immune system, signaling, senescence, phototransduction, and secretion (Supplementary Material 1, Table 2, and Figure 5). These results agree with the large repertoire of *CaM* target proteins known to play a role in ion homeostasis, metabolism, hormone biosynthesis, and gene expression, thereby promoting plant growth, development, stress response, and defense mechanisms (Ranty et al., 2006).

*CALMODULIN* is also associated with the WRKY family of transcription factors, which interacts with mitogen-activated protein kinase phosphatases (MPKs) that are responsible for activating the signaling cascades that mediate oxidative stress response, innate immunity, and response to cold, salinity, and drought (Rushton et al., 2010; Govardhana and Satyan, 2020). In this study, 10 upregulated unigenes were annotated as WRKY family members (Table 3), indicating the large role of this family has on transcription factors in *M. glaucescens* morphogenesis.

Recently, we have seen an increase in the number of studies exploring the advantages of *in vitro* culture for the production of secondary metabolites. *In vitro* tissue culture is known to increase the activity of metabolic pathways through the targeted application of nutrients, PGRs, light-induced factors, and elicitors of targeted downstream events (Kikowska et al., 2020). Some cacti are cultured *in vitro* to produce alkaloids and betalain-type pigments, demonstrating the potential these plants have to be sources of bioactive compounds for the pharmaceutical and food industries (Pérez-Molphe-Balch et al., 2015; Xie et al., 2020). The transcription factors of MYBs have already been demonstrated to be involved in the production of betalain in species like sugar beet and red pitaya (Hatlestad et al., 2015; Xi et al., 2019). In this study, MYBs expression was upregulated in the treated samples, suggesting a possible relationship between shoot organogenesis induction and betalain production.

In this study, shoot organogenesis induction positively affected the biosynthesis of phenylpropanoids, flavonoids, flavones, and flavonols (Supplementary Material 1 and Table 3). Moreover, treated samples exhibited the upregulation of several transcription factors associated with the biosynthesis of secondary metabolites (i.e., bHLH, MYB, NAC, WRKY, and YABBY) (Table 3) (Pal et al., 2018; Kayani et al., 2019; Xie et al., 2020). The biosynthetic pathways activated during shoot organogenesis induction of *M. glaucescens* indicate a strong potential for the production of bioactive compounds with a wide range of functions. Although the present findings could help optimize the micropropagation of *M. glaucescens* for horticultural and ornamental purposes and facilitate the conservation of endangered species and the biosynthesis of useful compounds, the results of this study are based on seed propagation. Therefore, some of the observed variations in organogenesis might be a result of genetic variability across samples. A previous study (Torres-Silva et al., 2018) has shown that clonal propagation is also correlated with somaclonal variation in *Melocactus*; thus, genetic variability within sexually or asexually generated populations could still be a confounding variable.

## Conclusion

The present study describes the assembly and functional annotation of the *de novo* transcriptome of *M. glaucescens*, a non-model plant with ornamental potential. This assembly was used to study gene expression and identify genes related to shoot organogenesis. The detection of numerous genes encoding putative transcription factors will benefit future studies on the regulation of gene expression during the growth and development of *M. glaucescens*. Altogether, this study expanded the existing genomic resources for cacti and provided a new platform for future genetic and biotechnological improvement of these plants for a variety of commercial purposes.

## Data Availability Statement

The data presented in the study are deposited in the NCBI Entrez repository (www.ncbi.nlm.nih.gov/bioproject/PRJNA663542), accession number PRJNA663542.

## Author Contributions

GT-S, AK, DB, SR, SS, CS, and WO conceived and designed the study. GT-S performed the experiments. GT-S and AK performed shoot organogenesis induction, sample collection, and RNA extraction. LC and DB performed RT-qPCR analyses. GT-S and SS performed *de novo* assembly pipelines. GT-S, SS, AA, and CS performed bioinformatics analyses. GT-S, DB, AK, SR, SS, AA, ER, DC, CS, and WO wrote the manuscript. All the authors read and approved the final manuscript.

## Conflict of Interest

The authors declare that the research was conducted in the absence of any commercial or financial relationships that could be construed as a potential conflict of interest.

## Publisher's Note

All claims expressed in this article are solely those of the authors and do not necessarily represent those of their affiliated organizations, or those of the publisher, the editors and the reviewers. Any product that may be evaluated in this article, or claim that may be made by its manufacturer, is not guaranteed or endorsed by the publisher.
