# Supplementary material for: Transcriptome Analysis of Melocactus glaucescens (Cactaceae) Reveals Metabolic Changes During in vitro Shoot Organogenesis Induction
Source: Front Plant Sci. 2021 Aug 20;12:697556. doi: 10.3389/fpls.2021.697556 (PMC8417902; doi:10.3389/fpls.2021.697556)
Supplement: Supplementary file 1 [file Data_Sheet_1.PDF]

Supplementary File 1. KEGG metabolic pathway categories among families of transcription factors in downregulated and upregulated unigenes during *Melocactus glaucescens de novo* shoot organogenesis induction. *indicates the same transcript in control and treated.

Metabolic pathway	Upregulated	Downregulated	Unigene shared in the pathway
Glycolysis / Gluconeogenesis	6	1	Fructose-bisphosphatealdolase, class I
Citrate cycle (TCA cycle)	3		
Pentose phosphate pathway	1	1	Fructose-bisphosphatealdolase, class I
Pentose and glucuronate interconversions	3	1*	
Fructose and mannose metabolism	2	1* and 1	Fructose-bisphosphatealdolase, class I
Galactose metabolism	1		
Ascorbate and aldarate metabolism	3		
Fatty acid biosynthesis	1	1	Acetyl-CoA carboxylase / biotin carboxylase 1
Fatty acid elongation	1		
Fatty acid degradation	5		
Steroid biosynthesis	4		
Ubiquinone and other terpenoid-quinone biosynthesis	1		
Oxidative phosphorylation	9	1* and 5	F-type H^+-transporting ATPase subunit a
Photosynthesis		15	
Photosynthesis - antenna proteins		5	
Arginine biosynthesis	1		
Purine metabolism	3		
Pyrimidine metabolism	1	1*	
Alanine, aspartate and glutamate metabolism	1	1*	
Glycine, serine and threonine metabolism	4	3*	
Cysteine and methionine metabolism	4		
Valine, leucine and isoleucine degradation	3		
Lysine degradation	1		
Arginine and proline metabolism	2		
Histidine metabolism	2		
Tyrosine metabolism	1	1*	

Phenylalanine metabolism	2	1*	
Tryptophan metabolism	2	1*	
Phenylalanine, tyrosine and tryptophan biosynthesis	2		
beta-Alanine metabolism	2		
Selenocompound metabolism	1		
Cyanoamino acid metabolism	1		
D-Glutamine and D-glutamate metabolism	1		
Glutathione metabolism	5	1	Glutathione S-transferase
Starch and sucrose metabolism	5		
Amino sugar and nucleotide sugar metabolism	5		
Streptomycin biosynthesis	1		
Glycerolipid metabolism	1	1*	
Inositol phosphate metabolism	3		
Glycerophospholipid metabolism	2	1*	
Ether lipid metabolism	2	1*	
Arachidonic acid metabolism		1	
Linoleic acid metabolism		1	
alpha-Linolenic acid metabolism	4	1*	
Sphingolipid metabolism	2		
Pyruvate metabolism	6	1	Acetyl-CoA carboxylase / biotin carboxylase 1
Chloroalkane and chloroalkene degradation	1		
Glyoxylate and dicarboxylate metabolism	3	1* and 3	Glycine dehydrogenase
Propanoate metabolism	3	1	Acetyl-CoA carboxylase / biotin carboxylase 1
One carbon pool by folate	1	1*	
Methane metabolism	3	1* and 1	Fructose-bisphosphatealdolase, class I
Carbon fixation in photosynthetic organisms	3	1* and 1	Fructose-bisphosphatealdolase, class I
Carbon fixation pathways in prokaryotes	5	1	Acetyl-CoA carboxylase / biotin carboxylase 1
Riboflavin metabolism	1		
Vitamin B6 metabolism	1		
Folate biosynthesis		1	

Limonene and pinene degradation	1		
Sesquiterpenoid and triterpenoid biosynthesis	1		
Nitrogen metabolism	3		
Sulfur metabolism	2		
Phenylpropanoid biosynthesis	6		
Flavonoid biosynthesis	3		
Flavone and flavonol biosynthesis	1		
Stilbenoid, diarylheptanoid and gingerol biosynthesis	1		
Metabolism of xenobiotics by cytochrome P450	1	1	Glutathione S-transferase
Insect hormone biosynthesis	1		
Drug metabolism - cytochrome P450	2	1	Glutathione S-transferase
Drug metabolism - other enzymes	2	1	Glutathione S-transferase
Biosynthesis of unsaturated fatty acids	2		
EGFR tyrosine kinase inhibitor resistance	3		
Antifolate resistance	1		
Platinum drug resistance	2	2	Solute carrier family 31 and Glutathione S-transferase
Quorum sensing	3		
Biofilm formation - *Escherichia coli*	1		
Ribosome	15	5*	
RNA transport	9		
mRNA surveillance pathway	2		
RNA degradation	1		
Spliceosome	10		
Proteasome	6		
Protein export	2		
PPAR signaling pathway	3		
MAPK signaling pathway	1		
MAPK signaling pathway - yeast	3		
MAPK signaling pathway - fly	1		
Ras signaling pathway	4	1*	

Rap1 signaling pathway	1		
MAPK signaling pathway - plant	8		
Calcium signaling pathway	2		
cGMP-PKG signaling pathway	2		
cAMP signaling pathway	3		
Chemokine signaling pathway	1		
HIF-1 signaling pathway	2		
FoxO signaling pathway	5		
Phosphatidylinositol signaling system	2		
Sphingolipid signaling pathway	4		
Phospholipase D signaling pathway	1	1*	
Plant hormone signal transduction	9	1	Small auxin upregulated RNAs
Cell cycle	3		
Cell cycle - yeast	2		
Meiosis - yeast	1		
Oocyte meiosis	4		
p53 signaling pathway	2		
Ubiquitin mediated proteolysis	5	1*	
Sulfur relay system	1		
Autophagy - animal	2		
Protein processing in endoplasmic reticulum	11	2*	
Lysosome	6	1	ATPeV
Endocytosis	5	1*	
Phagosome	9	2* and 1	v-ATPase and Tubulin alpha
Peroxisome	5	1* and 1	Superoxide dismutase
mTOR signaling pathway	7		
PI3K-Akt signaling pathway	8		
AMPK signaling pathway	2		
Apoptosis	4	1	Tubulin alpha
Longevity regulating pathway	3		

Longevity regulating pathway - worm	3		
Longevity regulating pathway - multiple species	3	1	Superoxide dismutase
Necroptosis	6		
Cellular senescence	4		
Cardiac muscle contraction		2	
Adrenergic signaling in cardiomyocytes	2		
Vascular smooth muscle contraction	1	1*	
Wnt signaling pathway		1	
Notch signaling pathway	1	1*	
TGF-beta signaling pathway	1		
Axon guidance	2		
Apelin signaling pathway	3		
Osteoclast differentiation	1		
Hippo signaling pathway	1		
Hippo signaling pathway - fly	3		
Hippo signaling pathway - multiple species	1		
Focal adhesion	1		
Tight junction	2	1	Tubulin alpha
Gap junction	1	1	Tubulin alpha
Antigen processing and presentation	6		
NOD-like receptor signaling pathway	2		
C-type lectin receptor signaling pathway	1		
Plant-pathogen interaction	4	1	Calmodulin
IL-17 signaling pathway	1		
Th17 cell differentiation	1		
Fc gamma R-mediated phagocytosis	2		
Circadian rhythm - plant	3	1*	
Circadian entrainment	2		
Thermogenesis	5	2	NADH-ubiquinone oxidoreductase chain 1
Long-term potentiation	1		

Synaptic vesicle cycle	4	2	ATPase
Neurotrophin signaling pathway	2	1*	
Retrograde endocannabinoid signaling	4		
Glutamatergic synapse	2		
Cholinergic synapse	1		
Serotonergic synapse	1		
GABAergic synapse	1		
Dopaminergic synapse	3		
Long-term depression	1		
Olfactory transduction	2		
Phototransduction	2		
Phototransduction - fly	1		
Inflammatory mediator regulation of TRP channels	1		
Regulation of actin cytoskeleton	2		
Insulin signaling pathway	3		
GnRH signaling pathway	2		
Progesterone-mediated oocyte maturation	2		
Estrogen signaling pathway	3		
Melanogenesis	1		
Thyroid hormone synthesis	1		
Thyroid hormone signaling pathway	1		
Oxytocin signaling pathway	2		
Glucagon signaling pathway	2		
Renin secretion	2		
Aldosterone synthesis and secretion	1		
Relaxin signaling pathway	1		
Parathyroid hormone synthesis, secretion and action	1		
Type II diabetes mellitus	1		
Insulin resistance	1		
Non-alcoholic fatty liver disease (NAFLD)	3	2*	

Cushing syndrome	1		
Vasopressin-regulated water reabsorption	1		
Proximal tubule bicarbonate reclamation	1		
Collecting duct acid secretion	4	2	ATPase
Salivary secretion	1		
Gastric acid secretion	1		
Pancreatic secretion		1	
Fat digestion and absorption		1	
Mineral absorption	2	1	Solute carrier family 31
Alzheimer disease	5	3*	
Parkinson disease	6	3*	
Amyotrophic lateral sclerosis (ALS)	1		
Huntington disease	6	3*	
Prion diseases		1	
Amphetamine addiction	1		
Morphine addiction	1		
Alcoholism	5		
Vibrio cholerae infection	5	1* and 2	ATPeV
Epithelial cell signaling in Helicobacter pylori infection	4	2	ATPeV
Pathogenic *Escherichia coli* infection	2	1	Tubulin alpha
Pertussis	2		
Legionellosis	3	1*	
Chagas disease (American trypanosomiasis)	2		
Toxoplasmosis	2		
Amoebiasis	1		
Tuberculosis	3	1	v-ATPase
Hepatitis C	3		
Hepatitis B	2		
Measles	2		

Human cytomegalovirus infection	3		
Influenza A	1		
Human papillomavirus infection	8	1* and 2	v-ATPase
Human T-cell leukemia virus 1 infection	6		
Kaposi sarcoma-associated herpesvirus infection	2		
Herpes simplex infection	4		
Epstein-Barr virus infection	7		
Human immunodeficiency virus 1 infection	4		
Pathways in cancer	7	1	Glutathione S-transferase
Transcriptional misregulation in cancer	2		
Viral carcinogenesis	9		
Chemical carcinogenesis	1	1	Glutathione S-transferase
Proteoglycans in cancer	2		
MicroRNAs in cancer	1		
Endometrial cancer	1		
Glioma	2		
Prostate cancer	3		
Melanoma	1		
Small cell lung cancer	3		
Breast cancer	1		
Hepatocellular carcinoma	2	1	Glutathione S-transferase
Gastric cancer	1		
Choline metabolism in cancer	1		
Systemic lupus erythematosus	3		
Rheumatoid arthritis	5	1* and 1	ATPase
Viral myocarditis	1		
Fluid shear stress and atherosclerosis	3	1	Glutathione S-transferase
Central carbon metabolism in cancer	2		

Supplementary File 2. Gene descriptions of down and upregulated genes annotated by BLAST results obtained by searching against the NCBI nr database

#	DOWNREGULATED
1	Chlorophyll a-b binding of LHCII type 1-like
2	Copper transporter 6-like
3	Conserved hypothetical protein
4	rRNA intron-encoded homing
5	Chloroplast photosystem II
6	EG5651
7	Ammonium transporter 1 member 3
8	V-type proton ATPase subunit e1
9	Thiosulfate sulfurtransferase chloroplastic
10	Probable stress-associated endoplasmic reticulum
11	Chlorophyll a-b binding chloroplastic
12	Arabinogalactan peptide 20-like
13	Photosystem I reaction center subunit IV chloroplastic
14	Photosystem II core complex s chloroplastic
15	Oligopeptide transporter 3
16	Chlorophyll a-b binding chloroplastic
17	Hypothetical protein GLYMA_02G283200
18	Metal transporter Nramp5
19	Early light-induced chloroplastic
20	D-glycerate 3- chloroplastic
21	Photosystem I reaction center subunit chloroplastic
22	Aquaporin SIP1-1-like
23	Predicted protein, partial
24	L-idonate 5-dehydrogenase
25	Transmembrane 256 homolog
26	V-type proton ATPase 16 kDa proteolipid subunit
27	Hypothetical protein AMTR_s03506p00001230
28	Aquaporin PIP2-1-like
29	Outer envelope membrane 7
30	Oxygen-evolving enhancer chloroplastic
31	Hypothetical protein PHAVU_011G146200g
32	Metal transporter Nramp5

33 Zinc finger family
34 50S ribosomal chloroplastic
35 PTB domain-containing engulfment adapter 1 isoform 1
36 50S ribosomal chloroplastic
37 Hypothetical protein SOVF_044150
38 E3 ubiquitin- ligase RHF2A isoform X1
39 Chlorophyll a-b binding chloroplastic
40 60S ribosomal L18a
41 LOB domain-containing 37
42 Chloroplast photosystem II
43 Small EDRK-rich factor 2
44 Superoxide dismutase [Cu-Zn]
45 CURVATURE THYLAKOID chloroplastic
46 Metal transporter Nramp5
47 Ribosomal L18p L5e family
48 10 kDa chaperonin
49 Oxoglutarate iron-dependent dioxygenase
50 DUF538 domain-containing
51 Transcription factor TGA6-like
52 Cytochrome b-c1 complex subunit 7
53 PREDICTED: uncharacterized protein LOC107806770
54 ORF137 (chloroplast)
55 Plastocyanin
56 CASP 2D1
57 STS14
58 DETOXIFICATION 40
59 ORF40s (chloroplast)
60 Hypothetical protein MANES_15G083600
61 Hypothetical protein SOVF_018990
62 Magnesium transporter 2 isoform 2
63 ORF137 (chloroplast)
64 Pollen Ole e 1 allergen and extensin family
65 NADH dehydrogenase [ubiquinone] 1 beta subcomplex subunit 3-B
66 E3 ubiquitin- ligase AIP2
67 Hypothetical protein LR48_Vigan04g102600

68 Oxygen-evolving enhancer chloroplastic
69 PetM of cytochrome b6 f complex subunit 7
70 Hypothetical protein SOVF_043810
71 PREDICTED: uncharacterized protein LOC105976406
72 Hypothetical protein M569_00222, partial
73 UPF0426 chloroplastic
74 Transmembrane 147
75 Cell wall-associated hydrolase
76 Tonoplast dicarboxylate transporter
77 Glycine cleavage system H mitochondrial
78 Hypothetical_ (chloroplast)
79 60S ribosomal L36-2
80 Oxygen-evolving enhancer chloroplastic
81 Sedoheptulose-1,7- chloroplastic
82 V-type proton ATPase 16 kDa proteolipid subunit
83 Magnesium transporter 2 isoform 2
84 Mitochondrial dicarboxylate tricarboxylate transporter DTC
85 Uncharacterized hydrolase -like
86 B-box domain 31-like
87 NADH dehydrogenase [ubiquinone] 1 beta subcomplex subunit 3-B
88 PRA1 family E
89 E3 ubiquitin- ligase RNF185
90 V-type proton ATPase subunit e1
91 Chlorophyll a-b binding chloroplastic
92 Probable pectinesterase pectinesterase inhibitor 34
93 DETOXIFICATION 40
94 ATP synthase CF0 B subunit (chloroplast)
95 Vacuolar iron transporter homolog 4
96 50S ribosomal chloroplastic
97 Senescence regulator S40
98 Photosystem I reaction center subunit chloroplastic
99 Ethylene-responsive transcription factor ERF011-like
100 Uncharacterized LOC100776314
101 Glutathione S-transferase
102 Cytochrome c biogenesis B (mitochondrion)

103 Cytochrome c oxidase subunit 2 (mitochondrion)

104 Uncharacterized LOC101244341

105 AF334834_1 chaperon

106 Metal transporter Nramp5

107 Plastoglobulin- chloroplastic

108 HEAT repeat-containing 8

109 Presenilin At1g08700

110 CONSTANS-like 5

111 Auxin-induced 6B

112 Snakin-2 isoform X1

113 Glycine-rich 2

114 Vacuolar sorting-associated 55 homolog

115 40S ribosomal S28

116 Hypothetical protein SOVF_205700

117 Aquaporin TIP1-1

118 bZIP transcription factor 53

119 DNA-binding S1FA

120 Transcription factor TCP7

121 Transcription factor TCP7

122 Ubiquitin-conjugating enzyme E2 7

123 ORF58e (chloroplast)

124 Aquaporin PIP2-1-like

125 PREDICTED: uncharacterized protein LOC104905519

126 CURVATURE THYLAKOID chloroplastic

127 Rubredoxin domain-containing

128 Fructose-bisphosphate aldolase chloroplastic

129 Uncharacterized protein LOC105635847

130 Cytochrome b6-f complex iron-sulfur chloroplastic

131 Peptidyl-prolyl cis-trans isomerase chloroplastic

132 Aquaporin SIP1-1-like

133 LOB domain-containing 37

134 Methylesterase 17

135 Probable steroid-binding 3

136 Rubredoxin family

137 Acyl carrier chloroplastic

Stress enhanced chloroplastic
Probable plastid-lipid-associated chloroplastic
CURVATURE THYLAKOID chloroplastic
Conserved hypothetical protein
CURVATURE THYLAKOID chloroplastic
Hypothetical protein B456_003G156600, partial
Plant F18G18-20
Endo-1,3 1,4-beta-D-glucanase
Photosystem I subunit O
Hypothetical protein EUGRSUZ_J02069
RING-H2 finger ATL16-like
Photosystem I reaction center subunit chloroplastic
Transmembrane ascorbate ferrireductase 1
Photosystem II M (chloroplast)
Chlorophyll a-b binding chloroplastic
Lipid-transfer DIR1
RING-H2 finger ATL57-like
Programmed cell death 4-like
Hydrogen peroxide-induced
CASP 5A2
Stress enhanced chloroplastic
PREDICTED: uncharacterized protein LOC108473348
PREDICTED: uncharacterized protein LOC104888529
Glyceraldehyde-3-phosphate dehydrogenase chloroplastic
Sucrose transport -like
Tobamovirus multiplication 2A-like
50S ribosomal chloroplastic
Dicarboxylate transporter chloroplastic
3-hexulose-6-phosphate isomerase-like
Oxalate-- ligase-like
Hypothetical protein SOVF_133900
Acetyl- carboxylase beta subunit (chloroplast)
RING FYVE PHD zinc finger superfamily isoform 1
Ribonuclease 1
Hypothetical protein SOVF_088820

173	Zinc finger A20 and AN1 domain-containing stress-associated 4-like
174	Photosystem II 5 kDa chloroplastic
175	Hypothetical protein SOVF_205700
176	La-related 6 isoform 1
177	Small GTPase superfamily
178	Copper transporter 1-like
179	Pre translocase subunit SECE1
180	Stem-specific TSJT1
181	Stem-specific TSJT1
182	Multi -bridging factor 1c
183	Serine--glyoxylate aminotransferase
184	ATP synthase gamma chloroplastic
185	Macrophage migration inhibitory factor homolog
186	Photosystem I reaction center subunit chloroplastic
187	WAS WASL-interacting family member 1 isoform X1
188	Probable carboxylesterase 12
189	Synechocystis YCF37
190	Bifunctional dihydrofolate reductase-thymidylate synthase-like isoform X1
191	Chlorophyll a-b binding chloroplastic
192	Cytochrome b6-f complex iron-sulfur chloroplastic-like
193	Aquaporin PIP1-3
194	Oil body-associated 1A
195	Phospholipase A2-alpha-like
196	E3 ubiquitin- ligase RHF2A isoform X1
197	Cell wall-associated hydrolase
198	Zinc finger family
199	mTERF domain-containing
200	Alpha tubulin 1
201	Plastocyanin
202	Ribulose bisphosphate carboxylase oxygenase activase chloroplastic isoform X1
203	Zinc finger GIS2
204	Probable calcium-binding CML36
205	RING FYVE PHD zinc finger superfamily isoform 1
206	Hypothetical protein BAE44_0001753
207	Uncharacterized N-acetyltransferase ycf52

	19 unigenes without Blast hit at NCBI nr database

#	**UPREGULATED**
1	Histone H2A
2	Serine threonine- kinase D6PK-like
3	Guanine nucleotide-binding subunit beta-2
4	Auxin response factor 2
5	Ran-binding 1 homolog a-like
6	Lipid transfer
7	LIGHT-DEPENDENT SHORT HYPOCOTYLS 4
8	Hyoscyamine 6-dioxygenase-like
9	MLP 43
10	Osmotin
11	Serine carboxypeptidase II-3-like
12	Probable flavin-containing monooxygenase 1
13	Purple acid phosphatase 3
14	Ripening-related 1
15	Trichome birefringence-like 36
16	GLUTAMINE DUMPER 3
17	Respiratory burst oxidase homolog B
18	Dual specificity phosphatase 1-like isoform X1
19	Calmodulin
20	Zinc finger CONSTANS-LIKE 16-like
21	BURP domain-containing 5
22	Basic 7S globulin-like
23	CHROMATIN REMODELING 4 isoform X1
24	Dehydration-responsive RD22
25	60S ribosomal L7-4
26	LIGHT-DEPENDENT SHORT HYPOCOTYLS 10-like
27	Arogenate dehydratase prephenate dehydratase chloroplastic-like
28	Exocyst complex component EXO70B1
29	LIGHT-DEPENDENT SHORT HYPOCOTYLS 10-like
30	Cryptochrome 1
31	Delta(12)-fatty-acid desaturase FAD2

32 Zinc finger 6-like
33 Transmembrane 9 superfamily member 1
34 60S ribosomal L17-2
35 Glycerophosphodiester phosphodiesterase GDPDL3-like
36 Xyloglucan galactosyltransferase XLT2
37 60S ribosomal L10a
38 TOPLESS
39 Calmodulin-binding 25
40 Transcription repressor OFP1-like
41 NUCLEAR FUSION DEFECTIVE 4-like
42 Non-specific phospholipase C2
43 Exocyst complex component SEC10
44 Leucine-rich repeat receptor-like serine threonine- kinase BAM1
45 Zinc finger CCCH domain-containing 40
46 GIGANTEA
47 Hypothetical protein BVRB_1g011250
48 Dirigent 22-like
49 EXORDIUM-like 3
50 Proteasome subunit alpha type-3
51 Kunitz trypsin inhibitor 2
52 Peroxidase 9
53 LIGHT-DEPENDENT SHORT HYPOCOTYLS 10-like
54 BAG family molecular chaperone regulator 7
55 BAG family molecular chaperone regulator 7
56 21 kDa -like
57 L-ascorbate oxidase
58 TORNADO 2
59 Copper transporter 5
60 Transmembrane 9 superfamily member 7
61 SIEVE ELEMENT OCCLUSION B
62 E3 ubiquitin- ligase KEG-like
63 Transmembrane 9 superfamily member 1
64 GPR107-like
65 Signal peptide peptidase
66 26S proteasome non-ATPase regulatory subunit 1 homolog A

67 60S ribosomal L3
68 Glucan endo-1,3-beta-glucosidase
69 Cytochrome P450 89A2
70 Ubiquitin-activating enzyme E1 1-like
71 Ferredoxin--nitrite chloroplastic
72 DUF506 domain-containing
73 Histone -like
74 FRIGIDA 3
75 tRNA-splicing ligase
76 26S proteasome non-ATPase regulatory subunit 13 homolog B
77 CBS domain-containing mitochondrial
78 Molybdate-anion transporter-like
79 Shaggy-related kinase eta
80 Argonaute 4
81 Aldehyde dehydrogenase family 2 member mitochondrial
82 Eukaryotic initiation factor 4A-3
83 Proteasome subunit beta type-5
84 TOM1 2
85 60S ribosomal L10a
86 Eukaryotic translation initiation factor
87 26S proteasome non-ATPase regulatory subunit 11 homolog
88 Expansin-A4
89 Expansin A10
90 Hypothetical protein SOVF_028230, partial
91 Hypothetical protein SOVF_014360
92 Thiosulfate 3-mercaptopyruvate sulfurtransferase mitochondrial isoform X2
93 Probable mannitol dehydrogenase
94 Eukaryotic translation initiation factor 3 subunit C
95 Delta(24)-sterol reductase
96 Phosphatidylcholine transfer
97 dnaJ homolog 2-like isoform X1
98 Endoglucanase 25-like
99 Lamin
100 Probable WRKY transcription factor 17
101 Ras GTPase-activating -binding 2

102 Serine threonine- kinase HT1-like
103 Threonine chloroplastic
104 Polygalacturonase inhibitor
105 ESCRT-related CHMP1B
106 Mitochondrial-processing peptidase subunit alpha-like
107 Eukaryotic translation initiation factor 3 subunit D-like
108 Chalcone isomerase
109 PHD finger ALFIN-LIKE 1-like
110 Cellulose synthase A catalytic subunit 3 [UDP-forming]
111 Cell division control 2 homolog A
112 SNW SKI-interacting -like
113 Senescence-associated family
114 Hypothetical protein BVRB_9g215280
115 60S ribosomal L9-1-like
116 Superoxide dismutase [Mn] mitochondrial
117 Nucleolin 1-like
118 Receptor kinase HAIKU2
119 Transmembrane 9 superfamily member 3
120 Ethylene-responsive transcription factor RAP2-12-like
121 Betaine aldehyde dehydrogenase chloroplastic
122 PLAT domain-containing 3-like
123 66 kDa stress
124 Probable carboxylesterase 18
125 S-adenosylmethionine decarboxylase
126 ATP synthase subunit mitochondrial
127 MLP 43
128 Polygalacturonase inhibitor
129 UDP-glucose 6-dehydrogenase 1
130 ETHYLENE INSENSITIVE 3-like 1
131 Plasma membrane ATPase 4
132 60S ribosomal L21-1
133 Tubby-like F-box 8
134 RING-H2 finger ATL56-like
135 Endoglucanase 6
136 ETHYLENE INSENSITIVE 3-like 1

137 VQ motif-containing family
138 Indole-3-acetic acid-amido synthetase
139 Ankyrin repeat-containing ITN1
140 Tubulin alpha-3 chain
141 Polygalacturonase-inhibiting
142 Abscisic acid receptor PYL4-like
143 UBP1-associated 2A-like
144 Probable serine threonine- kinase At4g35230
145 B2
146 SDE2 homolog
147 Cathepsin B-like
148 2-oxoglutarate-dependent dioxygenase DAO-like
149 Winged-helix DNA-binding transcription factor family
150 FRIGIDA 3
151 Phenylalanine ammonia-lyase
152 PREDICTED: uncharacterized protein LOC104889874
153 Polygalacturonase inhibitor
154 Dihydrolipoyl dehydrogenase mitochondrial
155 Octicosapeptide Phox Bem1p domain-containing family
156 Polygalacturonase inhibitor
157 Mitochondrial
158 Cytochrome P450 78A5-like
159 Sucrose synthase
160 3-ketoacyl- synthase 6
161 E3 ubiquitin- ligase XBAT31
162 60S ribosomal L19-3
163 Wound-induced 1-
164 Aldehyde oxidase GLOX-like
165 Eukaryotic translation initiation factor
166 Hypothetical protein BVRB_9g215280
167 Monodehydroascorbate reductase
168 dnaJ homolog
169 Lysine and serine-rich
170 Serine arginine-rich splicing factor RS40 isoform X1
171 ABC transporter I family member 19-like

172 Sesquiterpene synthase
173 Subtilisin-like protease
174 DEAD-box ATP-dependent RNA helicase 46
175 Cysteine ase RD21a-like
176 Glucan endo-1,3-beta-glucosidase 12
177 DUF868 domain-containing
178 Major allergen Pru ar 1
179 Pectinesterase
180 Pectinesterase-like
181 Histone H1
182 Aspartyl protease AED3
183 Elongation factor 2
184 Probable polygalacturonase
185 Methylenetetrahydrofolate reductase 2-like
186 Translation initiation factor IF-2-like
187 V-type proton ATPase subunit d2
188 Isocitrate dehydrogenase [NADP]
189 Transmembrane DDB_G0292058-like
190 Auxin-responsive SAUR32
191 Transcription elongation factor SPT4 homolog 2
192 Proteasome subunit alpha type-7
193 Zinc transporter 11
194 Gibberellin induced
195 Endoglucanase 1
196 60S ribosomal L7a
197 Polyol transporter 5
198 40S ribosomal S19-3
199 Aspartyl protease family 2
200 Abscisic stress-ripening 3
201 ATP-dependent Clp protease ATP-binding subunit homolog chloroplastic
202 VQ motif-containing 4
203 Beta-glucosidase 3B-like
204 Probable pectate lyase 5
205 Glucose-1-phosphate adenylyltransferase large subunit 1
206 Endoglucanase 17

207 Serine decarboxylase
208 Full=MJ-AMP1 Short=AMP1 Flags: Precursor
209 Non-specific lipid-transfer At2g13820
210 Plasminogen activator inhibitor 1 RNA-binding -like
211 Zinc finger CCCH domain-containing 20-like
212 BAHD acyltransferase DCR
213 Phosphatidylinositol 3,4,5-trisphosphate 3-phosphatase and -tyrosine-phosphatase PTEN2A
214 Elongation factor 2
215 Disulfide-isomerase
216 Zinc finger CCCH domain-containing 66
217 Probable polygalacturonase
218 T-complex 1 subunit epsilon
219 ATPase plasma membrane-type
220 Purple acid phosphatase 18
221 Polygalacturonase inhibitor
222 Polygalacturonase inhibitor
223 PREDICTED: uncharacterized protein LOC104898342
224 Vacuolar-processing enzyme
225 Pyruvate cytosolic isozyme
226 Kunitz trypsin inhibitor 2
227 60S ribosomal L17-2
228 40S ribosomal S8
229 C2 calcium-dependent membrane targeting
230 Cell division cycle 48 homolog
231 21 kDa seed
232 NUCLEAR FUSION DEFECTIVE 4
233 Phospholipase D alpha 1
234 Kunitz trypsin inhibitor 2
235 Probable acetyltransferase NATA1-like
236 Transcription factor DIVARICATA
237 Adenosine kinase 2
238 Probable pectate lyase 18
239 Probable fructokinase-4
240 Anti-fungal precursor
241 Lipoxygenase homology domain-containing 1-like

242 Alcohol dehydrogenase 1
243 Translocase of chloroplast chloroplastic
244 Nuclear receptor subfamily 4 group A member 3-like
245 NADH dehydrogenase subunit 9 (mitochondrion)
246 Glyoxysomal fatty acid beta-oxidation multifunctional MFP-a
247 Patellin-3
248 Beta-adaptin A
249 Pre-mRNA-processing-splicing factor 8A
250 NRT1 PTR FAMILY -like
251 Endogenous alpha-amylase subtilisin inhibitor-like
252 Aconitate cytoplasmic
253 Xyloglucan endotransglucosylase hydrolase 2
254 Wrky transcription factor 19
255 Cell wall vacuolar inhibitor of fructosidase 1
256 Purple acid phosphatase 22-like
257 UBP1-associated 2A
258 Hypothetical protein SOVF_055620
259 Auxin-responsive SAUR32
260 Probable metal-nicotianamine transporter YSL6
261 Eukaryotic translation initiation factor 3 subunit C-like
262 Anthocyanidin 3-O-glucoside 2 -O-glucosyltransferase
263 ABC transporter G family member 6-like
264 Cysteine ase 15A-like
265 Annexin RJ4
266 Probable histone
267 60S ribosomal L9
268 3-ketoacyl- synthase 10
269 Actin-depolymerizing factor 2
270 Serine arginine-rich splicing factor RS2Z33-like isoform X1
271 Probable sugar phosphate phosphate translocator At5g25400
272 Polyol transporter 5
273 Aspartic ase
274 AT-hook motif nuclear-localized 1
275 Octicosapeptide Phox Bem1p family isoform 1
276 Hypothetical protein SOVF_179180

277 Eukaryotic translation initiation factor NCBP
278 GDSL esterase lipase 1-like
279 Non-specific lipid-transfer 2-like
280 Ankyrin repeat domain-containing 2B
281 NADH dehydrogenase [ubiquinone] iron-sulfur mitochondrial
282 Probable WRKY transcription factor 7
283 Cysteine synthase
284 FT-interacting 1
285 Carbonic anhydrase 2 isoform X1
286 Kunitz trypsin inhibitor 2
287 Lipid-transfer DIR1
288 Shaggy-related kinase theta
289 40S ribosomal S6
290 Neutral ceramidase
291 Elongation factor 1-beta-like
292 14-3-3 A
293 V-type proton ATPase subunit D
294 Puromycin-sensitive aminopeptidase isoform X1
295 PREDICTED: uncharacterized protein LOC101262513
296 Receptor kinase TMK1-like
297 Probable galactinol--sucrose galactosyltransferase 6 isoform X2
298 Phosphatidylinositol 4-kinase gamma 5-like
299 Leucine aminopeptidase 1-like
300 E3 ubiquitin- ligase PRT6-like
301 Heat shock cognate 70 kDa 2
302 Abscisic stress ripening
303 Iron-sulfur cluster assembly 1-like
304 IAP 1 isoform 1
305 Polygalacturonase inhibitor-like
306 Serine threonine- phosphatase 2A 65 kDa regulatory subunit A beta isoform
307 Phospholipase D alpha 1
308 Basic 7S globulin-like
309 S-adenosylmethionine decarboxylase proenzyme-like
310 PUTATIVE TYPE 1 MEMBRANE family
311 EXORDIUM-like 2

312 Alcohol dehydrogenase
313 Membrane steroid-binding 2-like
314 Catalase isozyme 2
315 NADP-dependent malic enzyme
316 Dihydrolipoyl dehydrogenase mitochondrial
317 Aquaporin TIP4-1
318 FAM10 family At4g22670
319 2-alkenal reductase (NADP(+)-dependent)-like
320 Eukaryotic translation initiation factor
321 Caffeic acid O-methyltransferase
322 Probable polygalacturonase
323 Inositol-3-phosphate synthase
324 R3H domain-containing 2
325 Low-temperature-induced cysteine ase
326 PLASMODESMATA CALLOSE-BINDING PROTEIN 3-like
327 Cold shock domain-containing 4-like
328 Biotin carboxyl carrier of acetyl- carboxylase chloroplastic-like
329 Ubiquitin-conjugating enzyme E2 22
330 BURP domain RD22
331 Glucose-induced degradation 8 homolog isoform X1
332 Cysteine ase RD21a-like
333 DEAD-box ATP-dependent RNA helicase 7
334 NEDD8-conjugating enzyme Ubc12
335 Phosphoenolpyruvate carboxylase
336 Extensin-2-like isoform X2
337 Pyrophosphate-energized membrane proton pump 3
338 Fasciclin-like arabinogalactan 2
339 Abscisic acid receptor PYL4
340 DNA polymerase epsilon catalytic subunit
341 DNA polymerase epsilon catalytic subunit
342 EIN3-binding F-box 1-like
343 Nascent polypeptide-associated complex subunit muscle-specific form
344 Pectinesterase pectinesterase inhibitor
345 Ras-related RABA1f
346 Extensin-2-like isoform X2

347 Probable E3 ubiquitin- ligase LOG2
348 Probable galactinol--sucrose galactosyltransferase 2
349 Probable NADH dehydrogenase [ubiquinone] 1 alpha subcomplex subunit mitochondrial
350 Histone
351 Probable pectate lyase 18
352 CS domain
353 Probable fructokinase-4
354 Cathepsin B
355 Elongation factor 2
356 dnaJ homolog subfamily B member 6
357 Ethylene-responsive transcription factor ERF071
358 DEAD-box ATP-dependent RNA helicase chloroplastic
359 V-type proton ATPase subunit B 2
360 Glutathione-specific gamma-glutamylcyclotransferase 2
361 Transport sec31
362 60S ribosomal L19-3-like
363 Avr9 Cf-9 rapidly elicited
364 Sterol 14-demethylase-like
365 Avr9 Cf-9 rapidly elicited
366 14 kDa proline-rich -like
367 Subtilisin-like protease
368 UDP-glucuronate 4-epimerase 1
369 PREDICTED: uncharacterized protein LOC8287412
370 Class I chitinase
371 Hypothetical protein SOVF_116410
372 DUF239 domain-containing DUF4409 domain-containing
373 Mitochondrial Rho GTPase 1
374 Maturase (mitochondrion)
375 Sugar transporter ERD6-like 16
376 Salt-induced hydrophilic
377 WD40-like beta propeller repeat family
378 Programmed cell death 4
379 Polygalacturonase inhibitor
380 21 kDa -like
381 Hypothetical protein BVRB_9g215280

382 V-type proton ATPase subunit a3
383 Pyruvate decarboxylase 2
384 Zinc finger CCCH domain-containing 53-like isoform X2
385 Cytochrome P450 CYP736A12-like
386 Kunitz-type trypsin inhibitor-like 1
387 Gibberellin-regulated 14
388 Hypothetical protein BVRB_7g159390
389 Octicosapeptide Phox Bem1p family
390 Polygalacturonase inhibitor-like
391 Glycine-rich RNA-binding GRP1A
392 Calnexin homolog
393 Endoglucanase 17
394 Extensin isoform X2
395 60S ribosomal L18a
396 Calcium-dependent kinase 26
397 14 kDa proline-rich -like
398 Oligouridylate-binding 1B-like
399 5-methyltetrahydropteroyltriglutamate--homocysteine methyltransferase
400 Auxin-binding ABP19a
401 Phenylalanine ammonia-lyase
402 GDSL esterase lipase APG
403 Ribonuclease TUDOR 1-like
404 Probable aquaporin NIP5-1
405 Hypothetical protein SOVF_195340
406 LIGHT-DEPENDENT SHORT HYPOCOTYLS 10-like
407 60S ribosomal L3
408 Probable galactinol--sucrose galactosyltransferase 2
409 S-adenosylmethionine decarboxylase proenzyme-like
410 Hypothetical protein SOVF_020010
411 WD repeat-containing 70
412 Fasciclin-like arabinogalactan 7
413 Sucrose synthase
414 Xyloglucan endotransglucosylase hydrolase 2
415 Glycine-rich RNA-binding mitochondrial
416 60S ribosomal L28-2-like

417 MOB kinase activator-like 1A
418 UDP-glucuronate 4-epimerase 6
419 Fructose-bisphosphate aldolase chloroplastic
420 MLP 31
421 Probable WRKY transcription factor 7
422 Actin-depolymerizing factor
423 Trans-cinnamate 4-monooxygenase
424 Cytochrome P450
425 Non-specific lipid-transfer
426 Aspartic ase CDR1-like
427 Heat shock cognate 80-like
428 Dirigent 22-like
429 Vacuolar sorting-associated 62
430 60S ribosomal L23A
431 BURP domain RD22
432 28 kDa chloroplastic-like
433 D-3-phosphoglycerate dehydrogenase chloroplastic-like
434 Tetratricopeptide-like helical
435 Allene oxide synthase
436 Allene oxide synthase
437 Phospho-2-dehydro-3-deoxyheptonate aldolase chloroplastic-like
438 Linoleate 13S-lipoxygenase 2- chloroplastic-like
439 V-type proton ATPase catalytic subunit A
440 Phosphoenolpyruvate
441 Lipoxygenase homology domain-containing 1-like
442 Guanosine nucleotide diphosphate dissociation inhibitor 2
443 21 kDa
444 Leucine-rich repeat receptor-like serine threonine- kinase BAM1
445 7-dehydrocholesterol reductase
446 F-box SKP2A
447 Transport Sec61 subunit alpha-like
448 Dof zinc finger -like
449 Translocase of chloroplast chloroplastic
450 Transport SEC23
451 ru large subunit-binding subunit alpha

452 Pyruvate cytosolic isozyme
453 EXORDIUM-like 2
454 Hypothetical protein SOVF_177770 isoform B, partial
455 Peroxidase 12
456 SNF1-related kinase regulatory subunit gamma-1
457 Shaggy-related kinase eta isoform X1
458 Ubiquitin domain-containing DSK2a-like
459 Vacuolar-sorting receptor 3
460 21 kDa
461 Casein kinase 1 HD16
462 Polyubiquitin isoform X1
463 Oligouridylate-binding 1B-like
464 Glutathione S-transferase U9
465 Thiol protease aleurain-like
466 Heavy metal-associated isoprenylated plant 26-like
467 Hypothetical protein BVRB_2g031060
468 SUPPRESSOR OF K(+) TRANSPORT GROWTH DEFECT 1
469 PREDICTED: uncharacterized protein LOC104895058
470 Anti-fungal precursor
471 30S ribosomal chloroplastic
472 YTH domain-containing family 1-like isoform X1
473 ADP,ATP carrier mitochondrial
474 21 kDa -like
475 Vegetative cell wall gp1
476 Ethylene-responsive transcription factor 4
477 Cysteine ase 15A-like
478 Copper transport ATX1-like isoform X2
479 Polygalacturonase inhibitor
480 Zinc finger A20 and AN1 domain-containing stress-associated 1-like
481 Probable polygalacturonase
482 Ras-related RHN1
483 40S ribosomal S4-3
484 Elongation factor 2
485 Chalcone synthase
486 Cycloartenol-C-24-methyltransferase

487 S-adenosylmethionine decarboxylase
488 Trypsin inhibitor 3
489 FRIGIDA 4a
490 Non-specific lipid-transfer
491 DELLA GAIP-B-like
492 Pollen Ole e 1 allergen and extensin family
493 Polygalacturonase inhibitor
494 Probable pectate lyase 18
495 Isoflavone reductase
496 Auxin-responsive IAA16
497 Auxin-responsive IAA1
498 F-box PP2-B10-like
499 AChain The Angstroms Structure Of Pokeweed Antiviral
500 54S ribosomal mitochondrial
501 AChain The Angstroms Structure Of Pokeweed Antiviral
502 Eukaryotic translation initiation factor 3 subunit A
503 Cysteine ase RD21A
504 Photosystem II 5 kDa chloroplastic
505 bZIP transcription factor 11-like
506 Vacuolar-processing enzyme
507 Cysteine synthase
508 Plasminogen activator inhibitor 1 RNA-binding
509 Polygalacturonase 1 beta 3
510 Inter-alpha-trypsin inhibitor heavy chain H3
511 Signal recognition particle receptor subunit alpha homolog
512 Uncharacterized TPR repeat-containing At1g05150-like
513 Cyclin-dependent kinase F-4
514 Glutamate dehydrogenase B
515 Cell division cycle 48 homolog
516 ITR2_OPUST ame: Full=Trypsin inhibitor 2 Short= 2
517 14 kDa proline-rich -like
518 PLAT domain-containing 3-like
519 PLAT domain-containing 3-like
520 PLAT domain-containing 3-like
521 Transport sec31

522 Non-specific lipid-transfer At5g64080
523 5-methyltetrahydropteroyltriglutamate--homocysteine methyltransferase
524 BRASSINOSTEROID INSENSITIVE 1-associated receptor kinase 1-like
525 ABC transporter I family member 17
526 Argonaute 1-like
527 Peroxisomal acyl-coenzyme A oxidase 1
528 26S proteasome non-ATPase regulatory subunit 1 homolog A
529 Stromal 70 kDa heat shock-related chloroplastic
530 tRNA modification GTPase
531 Pectinesterase -like
532 Probable acyl- dehydrogenase IBR3
533 1-aminocyclopropane-1-carboxylate oxidase homolog 1
534 Polyadenylate-binding RBP45-like isoform X2
535 Coatomer subunit beta -2 isoform X1
536 Elongation factor 1-
537 SDE2 homolog
538 Glutamate dehydrogenase B
539 Heat shock cognate 80
540 V-type proton ATPase catalytic subunit A
541 Aspartic ase
542 29 kDa ribonucleo chloroplastic-like
543 Sphinganine C4-monooxygenase 1
544 21 kDa -like
545 PREDICTED: uncharacterized protein LOC100266414 isoform X2
546 Callose synthase 3
547 Phospho ECPP44-like
548 Gap junction beta-4 isoform 1
549 Splicing factor 3B subunit 4
550 Tubulin alpha-3 chain
551 CBL-interacting serine threonine- kinase 6
552 Vacuolar-processing enzyme
553 ERAD-associated E3 ubiquitin- ligase component HRD3A
554 CBS domain-containing CBSX6
555 F-box only 6
556 Membrane steroid-binding 2

557	Transmembrane 9 superfamily member 7
558	Auxin-responsive SAUR72-like
559	Patellin-3
560	Histone H2B-like
561	Eukaryotic translation initiation factor 3 subunit I-like
562	PDI-like 1-1 isoform 2
563	GATA zinc finger domain-containing isoform 1
564	Calreticulin-like
565	Cytochrome P450 76AD1-like
566	Peroxisomal membrane PEX14 isoform X1
	25 unigenes without Blast hit at NCBI nr database
